# Molecular Survey and Genetic Diversity of *Bartonella* spp. in Small Indian Mongooses (*Urva auropunctata*) and Their Fleas on Saint Kitts, West Indies

**DOI:** 10.3390/microorganisms9071350

**Published:** 2021-06-22

**Authors:** Alex Mau, Ana Cláudia Calchi, Pedro Bittencourt, Maria Jose Navarrete-Talloni, Caroline Sauvé, Anne Conan, Marcos Rogério André, Patrick Kelly, Ananda Müller

**Affiliations:** 1 One Health Center for Zoonoses and Tropical Veterinary Medicine, Department of Biomedical Sciences, Ross University School of Veterinary Medicine, Basseterre, Saint Kitts and Nevis; mauauom@gmail.com (A.M.); pbittencourt@rossvet.edu.kn (P.B.); MNavarreteTalloni@rossvet.edu.kn (M.J.N.-T.); pkelly@rossvet.edu.kn (P.K.); 2 Laboratório de Imunoparasitologia, Departamento de Patologia, Reprodução e Saúde Única—Faculdade de Ciências Agrárias e Veterinárias-Universidade Estadual Paulista (FCAV/UNESP), Jaboticabal, São Paulo 14884-900, Brazil; ana.calchi@hotmail.com (A.C.C.); mr.andre@unesp.br (M.R.A.); 3 Faculté de Médecine Vétérinaire, Université de Montréal, 3190 Rue Sicotte, Saint-Hyacinthe, QC J2S 2M2, Canada; caroline.sauve.6@umontreal.ca; 4 Centre de Recherche en Santé Publique, 7101 Avenue du Parc, Montréal, QC H3N 1X9, Canada; 5 Centre for Applied One Health Research and Policy Advice, Jockey Club College of Veterinary Medicine and Life Sciences, City University of Hong Kong, Hong Kong 518057, China; ayconan@cityu.edu.hk; 6 Instituto de Ciencias Clinicas Veterinarias, Facultad de Ciencias Veterinarias, Universidad Austral de Chile, Valdivia 4090000, Chile

**Keywords:** *Ctenocephalides felis*, *Bartonella henselae*, ‘*Candidatus* Bartonella kittensis’, haplotypes, Herpestidae

## Abstract

This study aimed to molecularly survey and evaluate the genetic diversity of *Bartonella* spp. in mongooses and their fleas from St. Kitts. Spleen (*n* = 54), blood (*n* = 71), and pooled flea samples, all identified as *Ctenocephalides felis* (*n =* 53), were submitted to TaqMan real-time quantitative PCR (qPCR) targeting *Bartonella*-*nuoG* fragment (84 bp). Positive samples underwent further conventional PCR assays targeting five loci (*gltA*, *rpoB*, *fstZ*, *nuoG*, and ITS), subsequent sequencing, and phylogenetic and haplotype analyses. The overall occurrence of *Bartonella* spp. in mongooses and fleas was 51.2% (64/125 [95% CI (42.1–60.2%)]) and 62.3% (33/53) [95% CI (47.9–75.2%)]), respectively. From samples sequenced across the five loci, 50.8% (33/65) were identified as *Bartonella henselae*, 26.2% (17/65) were 96.74–99.01% similar by BLAST analysis to an unidentified *Bartonella* sp. previously reported in Japanese badgers (*Meles anakuma),* and 23.1% (15/65) were co-infected with both species. Nucleotide polymorphism analysis showed low diversity amongst haplotypes but did concur with phylogenetic analysis, placing the unidentified species in a separate clade from *B. henselae* by multiple mutational events. Our data confirms that mongooses and *Ctenocephalides felis* fleas collected from them are not only potential reservoirs for *B. henselae* but also a novel *Bartonella* sp. which we propose be called ‘*Candidatus* Bartonella kittensis’.

## 1. Introduction

*Bartonella* is a genus in the order Rhizobiales that contains fastidious, Gram-negative, hemotropic, pleomorphic bacteria that are typically transmitted through arthropods [1]. Hematophagous vectors include fleas, ticks, lice, and biting flies [2], however, transmission through scratches, bites, and contact with infected body fluids has also been suggested [3]. Considered today as a re-emerging zoonotic disease, over 45 recognized species and subspecies have been identified of which 17 have demonstrated zoonotic potential [3]. 

The prevalence of *Bartonella* is seemingly dependent on geographical location and the presence of associated arthropod vectors [4,5]. Following infection, *Bartonella* invades the endothelial cells, erythrocytes and, possibly, the monocyte-macrophage system of mammalian hosts, resulting in persistent bacteremia [1,6,7]. Though the domestic cat and dog serve as primary hosts for several of the *Bartonella* spp. [8,9,10], *Bartonella* has also been detected in various wildlife species, such as rodents [11], wild carnivores [12], and bats [13]. The species most found in wild carnivores are *B. henselae*, followed by *B. rochalimae*, *B. clarridgeiae*, and *B. vinsonii* subsp. *berkhoffii* [12]. 

The small Indian mongoose (*Urva auropunctata*), herein referred to as the mongoose, is a highly invasive, terrestrial carnivorous mammal from the Herpestidae family that was first introduced to St. Kitts in 1884 to control rodent and snake populations [14]. Native to the Middle East and Southern Asia, their introduction to many islands and their subsequent expansion have led to devastating effects on local fauna and flora [15]. Furthermore, their scavenging behaviors and co-habitation in human dwellings poses zoonotic risks, with studies demonstrating their capacity to act as carriers for Rabies [16], *Leptospira* spp. [17], *Salmonella* spp. [18], *Toxoplasma* spp. [19], *Campylobacter* spp. [20], and *Bartonella* spp. [21,22]. Studies from Okinawa (Japan) and Grenada (West Indies) have identified the mongoose as a carrier of *B. henselae* with a PCR prevalence of 15.9% (10/63) and 35.5% (18/51), respectively [21,22].

The literature pertaining to *Bartonella* in the federation of Saint Kitts and Nevis, West Indies is sparse. To the authors’ best knowledge, *Bartonella* was first reported in St. Kitts in stray cats (63% [ 60/95]) by conventional (c) PCR targeting the 16S–23S rRNA intergenic region (ITS) [23]. Subsequent sequencing revealed the presence of *B. henselae* and *B. clarridgeiae*. Additionally, pan-*Bartonella* FRET-qPCR and a *gltA*-based cPCR detected *Bartonella* spp. in cats (39.7%, [58/146]) and cattle (54.8%, [23/42]) from St. Kitts but not horses, sheep, or donkeys [24]. Studies of *Bartonella* in wildlife from the country have demonstrated *Bartonella* spp. DNA by PCR in 0 to 72% of bats (depending on bat species) and one pooled sample of mites [25]. Most recently, *B.* *henselae* DNA was detected by PCR in fleas from mongooses on St. Kitts (10.3%, [9/87]) [26]. In regions such as the Caribbean where mongooses and domestic cats share the same habitats, it is possible that mongooses can serve as potential reservoirs of infection for domestic cats by the shared ectoparasite, *Ctenocephalides felis* [22], or direct interactions. The aim of the present study was to molecularly survey mongooses and their fleas as carriers for *Bartonella* and to assess the genetic diversity of *Bartonella* spp. in St. Kitts, West Indies.

## 2. Materials and Methods

### 2.1. Ethics 

Animal capture and handling was approved by the Animal Use Ethics Committee of University of Montreal (CÉUA 19-Rech-1993 and 19-Rech-1945), and further endorsed by the Ross University School of Veterinary Medicine (RUSVM) Institutional Animal Care and Use Committee (IACUC #TSU7.24.19). 

### 2.2. Sampling 

The samples utilized in this study were collected for an unrelated studying aiming to assess the epidemiology of the highly invasive mongoose and factors affecting trapping success and depopulation strategies [27]. Sampling occurred in St. Kitts, located at 17.3434° N, 62.7559° W, West Indies. The island has a tropical marine climate and a surface area of 174 square kilometers. It consists of a central area with extinct volcanoes surrounded by agricultural land. The study site was a 0.5 km^2^ plot known to be populated with mongooses located in Saint Peter parish and consisting of subtropical dry forest.

#### 2.2.1. Post-Mortem Samples

Mongooses were captured in cages (Tomahawk Live Trap, Hazelhurst, IW, USA) in June 2019. Traps were baited with canned tuna in water and checked in the morning and late afternoon, daily. Captured mongooses were immobilized with Zoletil 100 (tiletamine and zolazepam 1:1; Virbac, Bury Saint-Edmunds, UK) at around 15–20 mg/kg intramuscularly before being euthanized via intra-cardiac injection of saturated potassium chloride (75–100 mg/kg) as per the American Veterinary Medical Association Guidelines [28]. Death was confirmed by cardiac auscultation. Mongoose carcasses were transported on ice to RUSVM for necropsy. A total of 54 spleens were aseptically collected and stored at −80 °C prior to DNA extraction. A total of 130 fleas were collected using a pair of fine tipped forceps from individuals at the time of necropsy. 

#### 2.2.2. Live Animal Samples

Animals were captured in August 2019 and January 2020, as above except that traps were baited daily and checked within 24 h. Animals were immobilized (Zoletil 100, Virbac, Bury Saint-Edmunds, UK, at a dose of 5 mg/kg intramuscularly), and bled by venipuncture of the cranial vena cava. After a Passive Integrated Transponder (PIT) tag (Biomark APT12 FDX_B, Boise, ID) was attached, and the animals recovered from sedation, they were released in the same trapping location. A total of 71 blood samples (0.5 to 1 cc of whole blood in EDTA) obtained from trapped mongooses were transported on ice to reach RUSVM within 5 h of collection and stored at −20 °C, until DNA extraction. 

### 2.3. Flea Identification, DNA Extraction/Purification, and Quantification

Fleas were briefly washed with 70% alcohol at the time of microscopic identification [29] and then pooled in 53 samples by mongoose (1–8 fleas per pool). The pools were subsequently dried at room temperature before freezing with liquid nitrogen and macerated with a plastic pestle. Similarly, the frozen spleens were thawed at room temperature and 10 mg portions were refrozen with liquid nitrogen and manually macerated with a plastic pestle. DNA was extracted from the macerated flea suspensions (*n* = 53), 10 mg of the macerated spleen suspensions (*n* = 54) and 200 µL aliquots of the whole blood samples in EDTA (*n* = 71) with E.Z.N.A Tissue DNA Kit (E.Z.N.A Tissue DNA Kit, Omega Bio-Tek, GA, USA) as per the manufacturer’s instructions (100 µL elution). 

DNA concentration (ng/µL) and purity were estimated using a NanoPhotometer (Implen© GmbH, Schatzbogen, München Germany). The 260/280 nm absorbance ratio (OD_260_/OD_280_) yielded an estimate for sample purity and ratios of 1.8 ± 0.2 were deemed pure. To minimize the negative impact of excessive DNA on qPCR efficiency [30]_._, spleen samples with a DNA concentration > 50 ng/µL were diluted with TE buffer to 50 ng/µL.

### 2.4. DNA Integrity

To verify the presence of amplifiable DNA, DNA templates from the spleen and whole blood were assayed with a conventional PCR (cPCR) targeting the mammalian endogenous gene that encodes for the interphotoreceptor retinoid-binding protein (*irbp*), using primers IRBPF and IRBPR as previously described [31] (Table 1). DNA templates extracted from fleas were subjected to cPCR targeting an endogenous region of the *C. felis* 18S rRNA using primers *Cf*18SF and *Cf*18SR, as previously described [32] (Table 1). Conventional PCRs were performed in a Mastercycler^®^ Nexus (Eppendorf^®^, Hamburg, Germany), using nuclease-free water (Thermo Scientific^©^, Waltham, MA, USA) as a negative control. 

### 2.5. Molecular Survey of Bartonella spp.

For the detection and quantification of *Bartonella*, all blood and spleen and *C. felis* positive for *irbp* and 18S rDNA-based PCR assays were submitted to a previously described quantitative real-time PCR (qPCR) targeting a fragment of the *Bartonella* spp. *nuoG* gene and capable of detecting as low as 10 copies of plasmid/reaction [33]. Amplifications were performed with final volumes of 10 µL, containing 5 µL of GoTaq™ Probe qPCR Master Mix 2x buffer (Promega Corporation^©^, Madison, WI, USA), 1.2 µM of each primer (F-Bart 5′-CAATCTTCTTTTGCTTCACC-3′ and R-Bart 5′-TCAGGGCTTTATGTGAATAC-3′) and hydrolysis probe (TexasRed-5′–TTYGTCATTTGAACACG-3′[BHQ2a-Q]), 1 µL of DNA template and 0.4 µL of sterile nuclease-free water (Thermo Scientific^©^, Waltham, MA, USA). qPCR assays were conducted on Low-Profile Multiplate™ unskirted PCR plates (BioRad^©^, Hercules, CA, USA) in a CFX96 thermal cycler (BioRad^©^, Hercules, CA, USA, following the MIQE (Minimum Information for Publication of Quantitative Real-Time PCR experiments) [34]. All qPCR were run in duplicate and amplification conditions were 95 °C for 3 min, followed by 40 cycles at 95 °C for 10 s and 52.8 °C for 30 s. Amplification efficiency (*E*) was calculated from the slope of the standard curve in each run using the following formula (*E* = 10^−1/slope^) [34]. Standard curves were constructed using 10-fold serial dilutions (2.0 × 10^7^ to 2.0 × 10^0^) of a gBlock^®^ (Integrated DNA Technologies, Coralville, IA, USA) encoding an 83 bp fragment of the *nuoG* gene of *B. henselae* (Integrated DNA Technologies, Coralville, IA, USA). The number of gBlock^®^ copies was determined according to the formula [Xg μL^−1^ DNA/(gBlock^®^ length (BP) × 660) × 6.022 × 10^23^ × gBlock^®^ copies μL^−1^]. *Bartonella henselae* DNA was used as a positive control [35]. All PCR runs were performed with nuclease-free water (Thermo Scientific^©^, Waltham, MA, USA) as a negative control. Replicates showing a Cq (quantification cycle) difference higher than 0.5 were retested. Duplicate samples with a Cq difference below 0.5 were considered “consistent” results. 

### 2.6. Molecular Characterization of Bartonella spp.

To facilitate further molecular characterization, all spleen/whole blood and flea pools positive for *Bartonella* spp. *nuoG*-based qPCR assay were subjected to cPCR assays targeting five loci (*gltA*, *rpoB*, *nuoG*, ITS, *fstZ*) (see Table 1). All cPCR assays were run in a Mastercycler^®^ Nexus (Eppendorf^®^, Hamburg, Germany) using sterile nuclease-free water (Thermo Scientific^©^, Waltham, MA, USA) as a negative control and *B. henselae* DNA as a positive control [35]. PCR products were separated with electrophoresis on a 1% agarose gel (UltraPure™ Agarose, Thermo Fischer Scientific^©^, Waltham, MA, USA) stained with SYBR Safe DNA gel stain (Thermo Fischer Scientific^©^, Waltham, MA, USA). 

Positive samples presenting bands with subjectively significant staining were enzymatically purified with Exonuclease I (Exo I) and Shrimp Alkaline Phosphatase (rSAP) as per the manufacturer’s instructions (New England Biolabs, Ipswich, MA, USA). All purified samples were sent to Macrogen (Geumcheon-gu, Seoul, South Korea) for automatic sequencing by Sanger’s method with ABI PRISM 310 DNA Analyzer (Applied Biosystems/Perkin-Elmer).

### 2.7. BLAST Analysis

Quality of obtained sequences was evaluated using Phred-Phrap version 23 [40,41] with Phred quality scores (peaks around each base call) established as higher than 20 (99% accuracy of the base call). The percentage of identities was obtained using nBLAST [42]. The similarity of the obtained sequences with those in GenBank was determined by percentage identity and E-value, and only the best hit (first search result) was used. 

### 2.8. Phylogenetic Analysis

Sequences obtained from this study and those in GenBank were aligned using MAFFT software version 7 [43]. jModelTest2 [44], via the CIPRES Science Gateway [45], was used to determine the best evolutionary model under the Akaike Information Criterion (AIC) [46]. Bayesian inference was selected for phylogenetic analysis, utilizing MrBayes 3.1.2 [47]. The Bayesian analysis was made with 10^6^ generations and several substitutions and the posterior probabilities with 10,000 repetitions, chains = 4, number of chains per microprocessor = 1, burn-in = 25%, and an average standard deviation of split less than 0.01. The phylogenetic trees were edited with Treegraph (2.0.56–381 beta) [48].

### 2.9. Haplotype Analysis (Genetic Diversity)

To assess genetic diversity, the sequences for *gltA*, *rpoB*, *fstZ*, *nuoG* and ITS were aligned with sequences available in GenBank. The software DnaSP v5 [49] was used to calculate nucleotide diversity (π), polymorphism level [haplotype diversity (Hd), number of haplotypes (h)], number of variable sites (VS), and the average number of nucleotide differences (K). Nucleotide sequences were submitted to the TCS network [50] and a Split-Network was created using popART [51]. 

## 3. Results

### 3.1. Amplifiable DNA, and Bartonella spp. Survey

Spleens from all 54 mongooses (mean DNA concentration prior to dilution = 333.51 ng/µL) and 71 blood samples (DNA concentration = 7.64 ng/µL) tested positive for the *irbp* mammalian endogenous gene.

All 130 fleas were individually morphologically identified by microscopy as *C. felis* and the 53 pooled flea samples (mean DNA concentration = 16.66 ng/µL) were positive for the *C. felis* endogenous region of 18S rDNA. 

One third (33.3%; 18/54 [95% CI (21.1–47.5%)]) of the spleen samples and 64.7% (46/71) [95% CI (52.5–75.8%)] of the whole blood samples were positive in the qPCR targeting the *Bartonella nuoG* gene. Overall, 51.2% (64/125) [95% CI (42.1–60.2%)] of the mongooses were positive for *Bartonella* spp. with the qPCR targeting the *nuoG* gene (Mean ± SD of reactions’ efficiency = 94.46% ± 4.44; *r*^2^ = 0.995 ± 0.003; slope = −3.499 ± 0.15; Y-intercept = 32.835 ± 3.076) (Table S1). Thirty-six (36/64) samples had consistent Cq values (Mean Cq = 31.51) and the quantification of *Bartonella* spp. ranged from 4.74 × 10^−2^ to 1.92 × 10^2^ *nuoG*-copies/μL (mean = 2.20 × 10), with higher concentrations observed in the blood (4.15 × 10^−1^ to 1.92 × 10^2^) when compared to spleens (4.74 × 10^−2^ to 1.15 × 10^0^). 

A high percentage of the pooled flea samples (62.3.2%; 33/53; [95% CI (47.9–75.2%)]) were positive for in the *nuoG* qPCR (Mean and SD of reaction’s efficiency = 95.13% ± 5.20; *r*^2^ = 0.994 ± 0.007; slope = −3.449 ± 0.15; Y-intercept = 35.18 ± 1.147). Twenty pooled (20/33) flea samples had consistent Cq values (Mean = 27.92 ± 4.46) and the quantification of *Bartonella* spp. ranged from 2.73× 10^−1^ to 6.55 × 10^3^ *nuoG*-copies/μL (mean ± SD = 1.13 × 10^3^ ± 2 × 10^3^). 

A total of 41/97 (spleen, blood, or flea pools) positive samples had inconsistent *Bartonella*-qPCR quantification assays, thus their Cq and quantification results are not reported in the present work.

### 3.2. Molecular Characterization and BLAST Analysis

Out of 97 *Bartonella* spp. *nuoG*-qPCR positive samples (blood, spleen, and pooled fleas), 95.9% (93/97) [95% CI (89.8–98.9%)] were also positive for at least one other of the genes we tested by cPCR: 92.9% (90/97) for *rpoB*, 87.6% (85/97) for *gltA*, 83.5% (81/97) for *nuoG*, 70.1% (68/97) for ITS and 65.0% (63/97) for *ftsZ* (Table S1).

A total of 209 amplicons from the mongooses and fleas obtained across the five loci were submitted for sequencing. Of these, 166 provided useable consensus sequences: 42 for *gltA*, 50 for *rpoB*, 31 for ITS, and 21 each for *ftsZ* and *nuoG.* The remaining 43 amplicons yielded poor quality forward or reverse sequences based on Phred-Phrap analysis, which precluded their further use. Obtained consensus sequences were deposited to the international database GenBank under the following accession numbers: MW728178-MW728269, MW743240-MW743270, MW748304-MW748345, MW767381. These sequences were compared using nBLAST on the 02/16/2021 and the best hits used to determine percentage identities as summarized in Table 2 (For extensive results see Tables S2–S4).

Overall, 166 sequences (across five loci) were retrieved from a total of 65 DNA samples (spleen, blood, and fleas). From this, 33/65 (50.8%) samples were identified by BLASTn as *B. henselae* and 17/65 (26.2%) as *Bartonella* sp. previously detected in *M. anakuma*. The remaining 15/65 (23.1%) samples were co-positive to both *B. henselae* and *Bartonella* sp. (9/15 blood, 4/15 fleas and 2/15 spleen) (Table 3).

### 3.3. Phylogenetic Analysis

**Figure 1:**
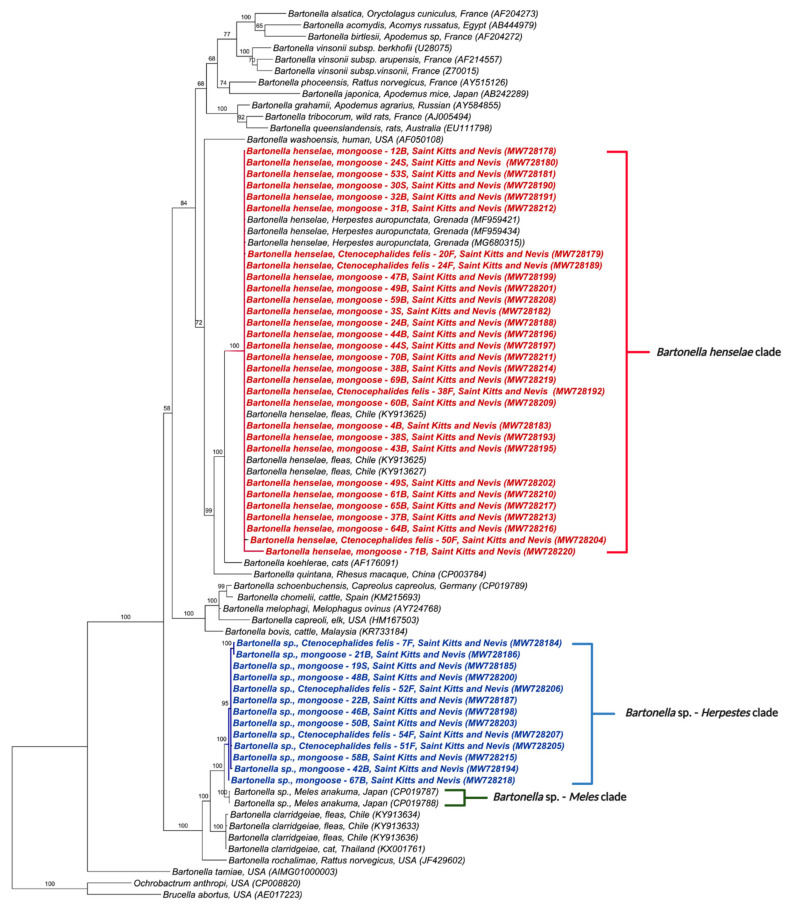
Phylogenetic analysis of *gltA* sequences (750 bp) based on the topology generated by the Bayesian analysis with the TIM3+I+G evolutionary model. Sequences from the present study are colored in red and blue. The numbers at the nodes correspond to posterior probabilities with 10,000 repetitions. *Brucella abortus* and *Ochrobactrum anthropi* were used as outgroups.

The phylogenetic tree inferred by Bayesian analysis based on the *Bartonella* spp. *gltA* gene placed the 42 obtained sequences into two distinct clades (Figure 1); one containing sequences from this study, *B. henselae* in mongooses from Grenada (MF959421, MF95934, MG680315) and fleas from Chile (KY913625, KY913627); and the other closely related to *Bartonella* sp. previously detected in *M. anakuma* from Japan (CP019787, CP019788) with 100% branch support (Figure 1). The closest identified species to this *M. anakuma* clade was *B. clarridgeiae.*

**Figure 2:**
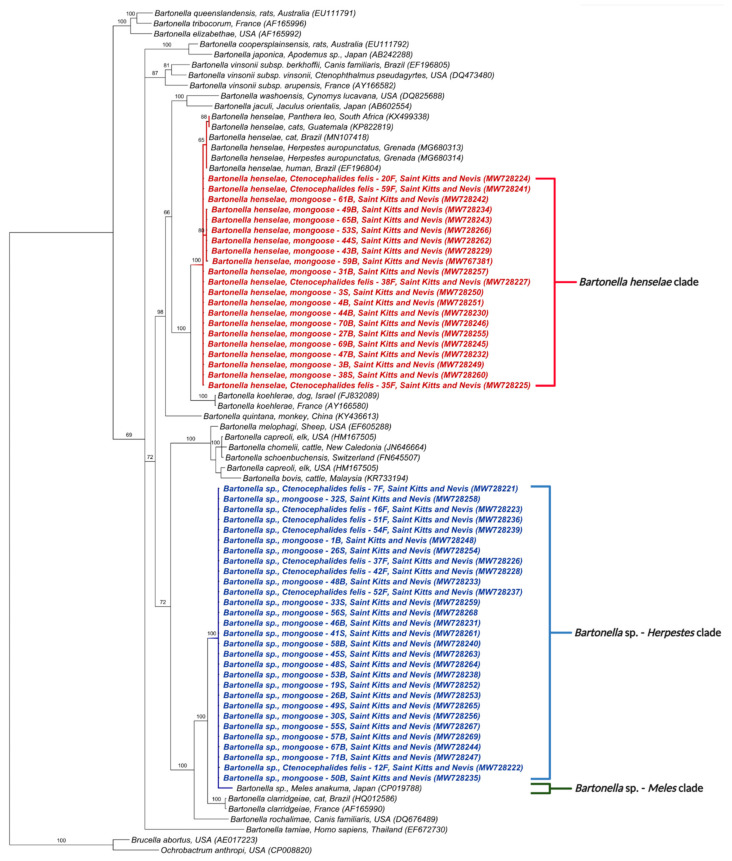
Phylogenetic analysis of *rpoB* sequences (333 bp) based on the topology generated by the Bayesian analysis with the TIM3+I+G evolutionary model. Sequences from the present study are colored in red and blue. The numbers at the nodes correspond to posterior probabilities with 10,000 repetitions. *Brucella abortus* and *Ochrobactrum anthropi* were used as outgroups.

A similar phylogenetic tree based on the *Bartonella* spp. *rpoB* gene also positioned the 50 obtained sequences into two distinct clades (Figure 2). One clustered the sequences from this study with *B. henselae* in lions from South Africa (KX499338), cats from Brazil and Guatemala (KP822819, MN107418), a human from Brazil (EF196804), and mongooses from Grenada (MG680313, MG6801314). The other clade, as above, included *Bartonella* sp. in *M. anakuma* from Japan (CP019788) with 100% branch support (Figure 2). 

**Figure 3:**
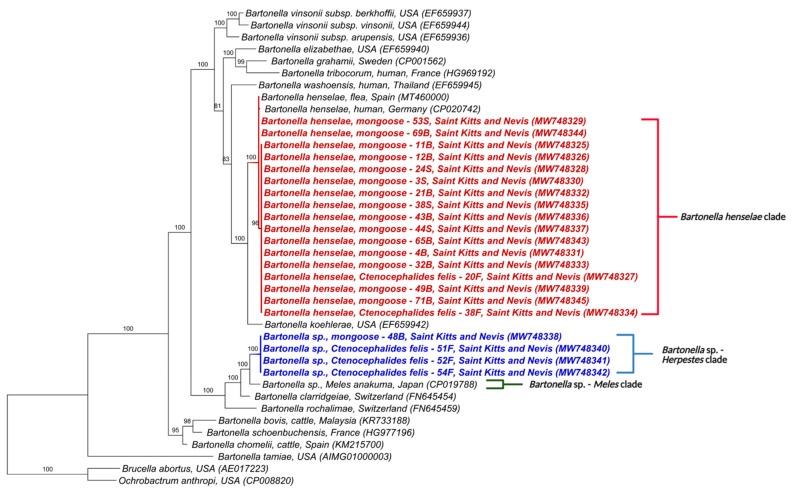
Phylogenetic analysis of *nuoG* sequences (400 bp) based on the topology generated by the Bayesian analysis with the TIM3+I+G evolutionary model. Sequences from the present study are colored in red and blue. The numbers at the nodes correspond to posterior probabilities with 10,000 repetitions. *Brucella abortus* and *Ochrobactrum anthropi* were used as outgroups.

**Figure 4:**
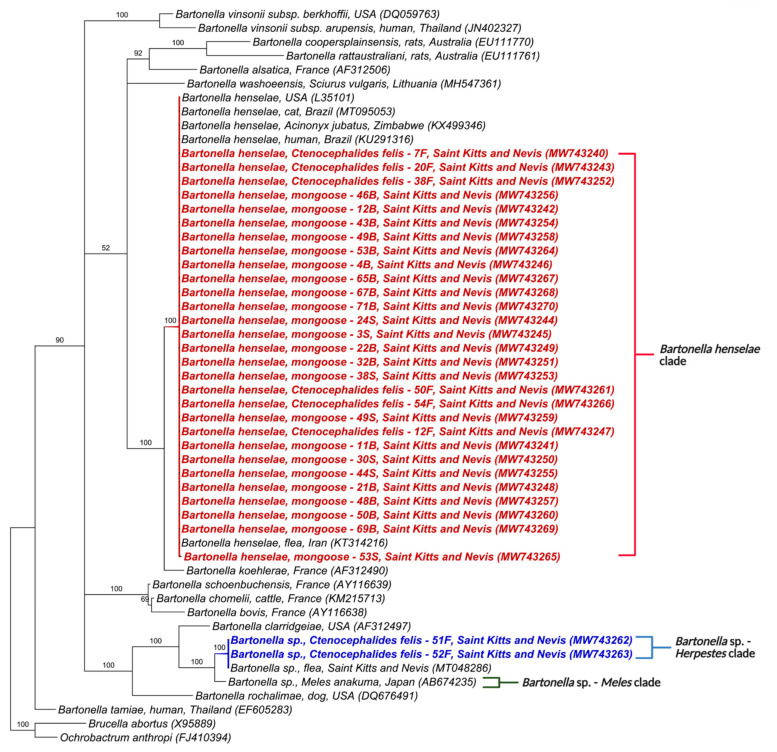
Phylogenetic analysis of 16–23S rRNA ITS sequences (453–717 bp) based on the topology generated by the Bayesian analysis with the TIM3+I+G evolutionary model. Sequences from the present study are colored in red and blue. The numbers at the nodes correspond to posterior probabilities with 10,000 repetitions. *Brucella abortus* and *Ochrobactrum anthropi* were used as outgroups.

Similarly, the phylogenetic trees for *nuoG* and ITS also formed two clades (Figure 3 and Figure 4). The first was composed of *B. henselae* and the other, similar to *gltA* and *rpoB,* included *Bartonella* sp. in *M. anakuma* from Japan (CP019788) with 100% branch support. In addition, the ITS phylogeny closely positioned the *Bartonella* sp. in mongooses to a previous *Bartonella* sp. detected in fleas from St Kitts (MT048286). 

In contrast, the phylogenetic tree for *ftsZ* clustered the obtained sequences into a single clade containing *B. henselae* sequences in a dog from China (JQ009431), human from Germany (CP020742) and cat from Guatemala (KP822811). 

**Figure 5:**
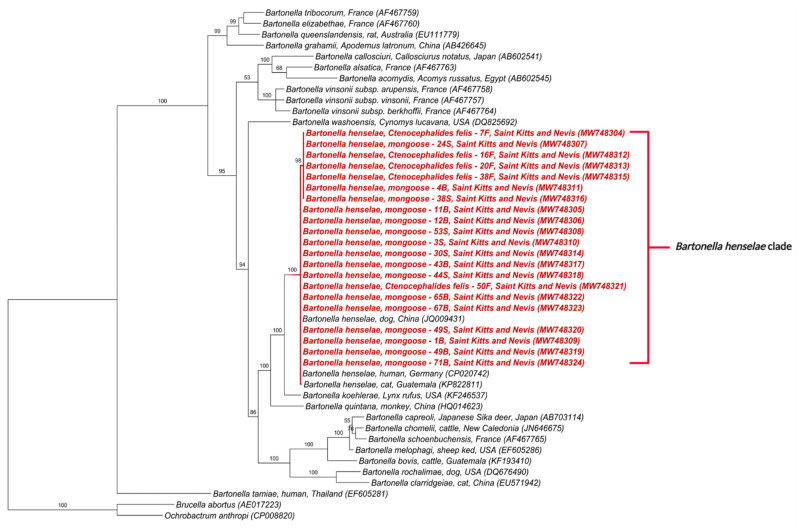
Phylogenetic analysis of *ftsZ* sequences (515 bp) based on the topology generated by the Bayesian analysis with the TIM3+I+G evolutionary model. Sequences from the present study are colored in red. The numbers at the nodes correspond to posterior probabilities with 10,000 repetitions. *Brucella abortus* and *Ochrobactrum anthropi* were used as outgroups.

In concordance with the BLASTn analyses, some samples that were co-positive (Tables S2–S4) clustered, in the phylogenetic inference, with two *Bartonella* species when more than one sequence corresponding to different target genes was available. Samples 30S, 49S, 1B, 21B, 22B, 46B, 48B, 50B, 53B, 67B, 71B, 7F, 12F, 16F, and 54F clustered with *B. henselae* in at least one gene and *Bartonella* sp. from *M. anakuma* in another gene (Figure 1, Figure 2, Figure 3, Figure 4 and Figure 5). 

### 3.4. Haplotype Analysis (Genetic Diversity)

**Figure 6:**
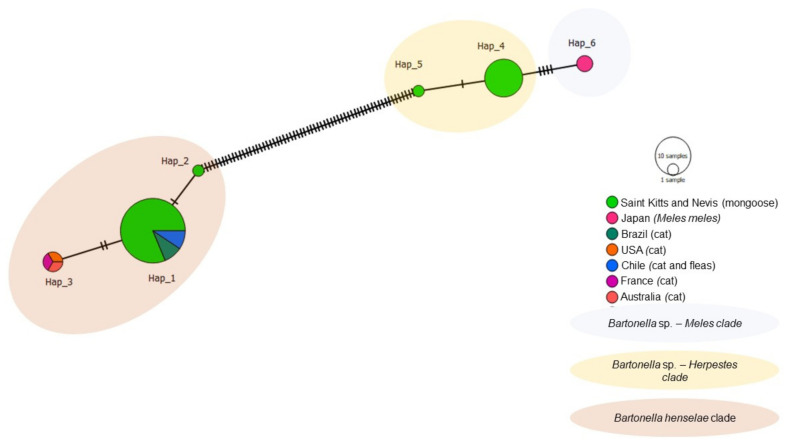
Haplotype network for *Bartonella gltA* sequences detected in mongooses and their fleas, Saint Kitts (haplotypes #1, #2, #4, and #5), combined with *Bartonella* sequences that were previously detected in cats, fleas, and *Meles* spp. worldwide. Each small dash represents mutations. The haplotype network was generated with DNAsp data followed by analysis in PopArt using geographic coordinates and a TCS network.

The haplotype analysis based on 50 *gltA Bartonella* sequences, including 39 obtained in the present study and 11 worldwide sequences from cats, fleas, and European badgers revealed six different haplotypes (Table 4, Figure 6). Haplotype #1 consisted of 26 sequences obtained in this study from mongooses and fleas and *B. henselae* sequences detected in cats and their fleas from Chile and Brazil. Haplotype #2 contained one *B. henselae* sequence obtained from a mongoose in this study. Haplotype #3 contained *B. henselae* sequences from Australia, USA, and France. Haplotype #4 comprised of *Bartonella* sp. sequences obtained from mongooses and fleas from this study. Haplotype #5 contained one *Bartonella* sp. sequence obtained from a mongoose in this study. Haplotype #6 encompassed *Bartonella* sp. sequences obtained from *M. anakuma* in Japan. Haplotype #4 and #5 from *Bartonella* sp. were exclusive to Saint Kitts and separated by a single mutational event (Figure 6), whereas haplotype #5 (*Bartonella* sp.) and #2 (*B. henselae*) from Saint Kitts were separated by many mutational events. Corroborating with the phylogenetic inferences, while haplotypes #4 and #5 formed a clade that was more closely related to *Bartonella* sp. haplotype #6 (*M. anakuma)*; haplotypes #1 and #2 formed a clade including *B. henselae* sequences from Chile and Brazil. 

**Figure 7:**
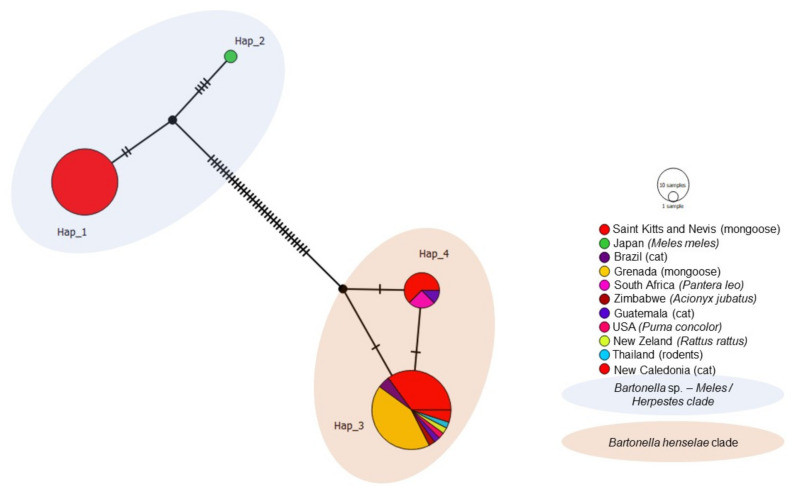
Haplotype network for *Bartonella rpoB* sequences detected in mongooses and their fleas, Saint Kitts (haplotypes #1, #3, and #4), combined with *Bartonella* sequences that were previously worldwide. Each small dash represents mutations. Dark circles represent median vectors. The haplotype network was generated with DNAsp data followed by analysis in PopArt using geographic coordinates and a TCS network.

Out of the 77 *Bartonella rpoB* analyzed sequences, including 47 obtained in the present study and 30 *Bartonella* previously detected in domestic and wild cats worldwide, rodents, and Japanese badger, four different haplotypes were obtained (Table 4, Figure 7). Haplotype #1 consisted of *Bartonella* sp. sequences from mongooses and fleas obtained in the present study and was not shared with other geographic locations. Haplotype #2 contained one *Bartonella* sp. sequence obtained from *M. anakuma* from Japan. Haplotype #3 was widely distributed and encompassed *B. henselae* sequences obtained from the present study, as well as cats (Brazil, Guatemala, New Caledonia), lions (South Africa), cheetahs (Zimbawe), mountain lions (USA), mongoose (Grenada), rats (New Zealand), and rodents (Thailand). Haplotype #4 consisted of *B. henselae* sequences obtained in the present study and previously detected in mongooses (Grenada), cats (Brazil), and lions (South Africa). *Bartonella* sp. haplotypes #1 and #2 formed a clade and arose from a common median vector which may reflect an unsampled sequence from extant species or extinct ancestral sequence (Figure 7). Similarly, *B. henselae* haplotypes #3 and #4 formed a clade and emerged from a different median vector, which is separated from the previous clade by many mutations. Haplotype network patterns followed the ones observed in the phylogenetic tree based on *rpoB*. 

**Figure 8:**
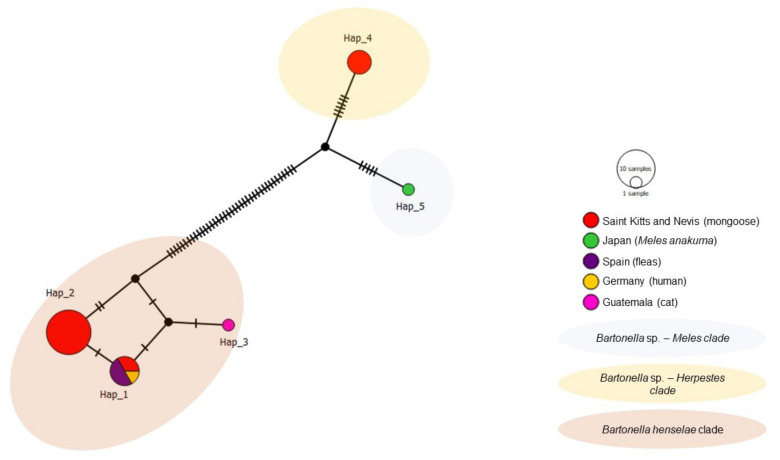
Haplotype network for *Bartonella nuoG* sequences detected in mongooses and their fleas, Saint Kitts (haplotypes #1, #2, and #4), combined with *Bartonella* sequences that were previously worldwide. Each small dash represents mutations. Dark circles represent median vectors. The haplotype network was generated with DNAsp data followed by analysis in PopArt using geographic coordinates and a TCS network.

The haplotype analysis of *nuoG* was based on 26 sequences (20 from the present study and six from various species worldwide) identified five haplotypes (Table 4, Figure 8). Haplotype #1 was comprised of *B. henselae* sequences from the present study (mongoose on St. Kitts), a human from Germany and fleas from Spain. Haplotypes #2 and #3 consisted of *B. henselae* from mongooses in the present study and a cat from Guatemala, respectively. Haplotypes #4 (mongoose from St. Kitts) and #5 (*M. anakuma* from Japan) arose from a common median vector. Haplotypes #2 and #4 were only detected in St. Kitts. 

**Figure 9:**
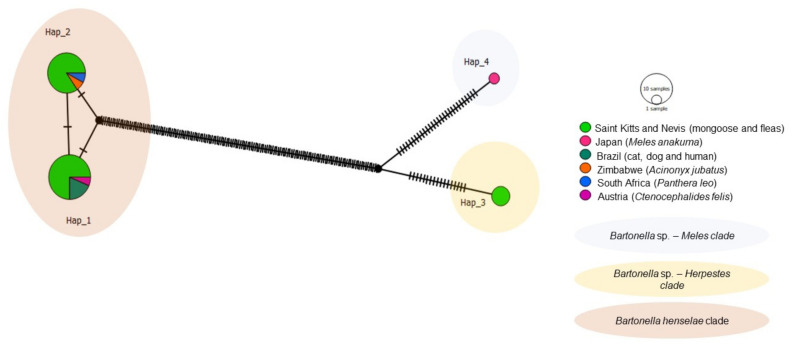
Haplotype network for *Bartonella* ITS sequences detected in mongooses and their fleas, Saint Kitts (haplotypes #1, #2, and #4), combined with *Bartonella* sequences that were previously worldwide. Each small dash represents mutations. Dark circles represent median vectors. The haplotype network was generated with DNAsp data followed by analysis in PopArt using geographic coordinates and a TCS network.

Four haplotypes were identified from the 33 sequences used for *Bartonella* ITS including 25 from this study and 8 from various animal species worldwide including cats, dogs, and humans from Brazil, cheetahs from Zimbabwe, lions from Africa, and a flea from Austria (Table 4, Figure 9). A common median vector gave rise to Haplotypes #1 and #2, which formed the *B. henselae* clade. This clade was separated by many mutations from another median vector giving rise to Haplotypes #3 (*Bartonella* sp. in mongoose and fleas from St. Kitts, not shared with other geographic regions) and Haplotype #4 (*Bartonella* sp. from *M. anakuma)*, both separated by a median vector and several mutational events.

**Figure 10:**
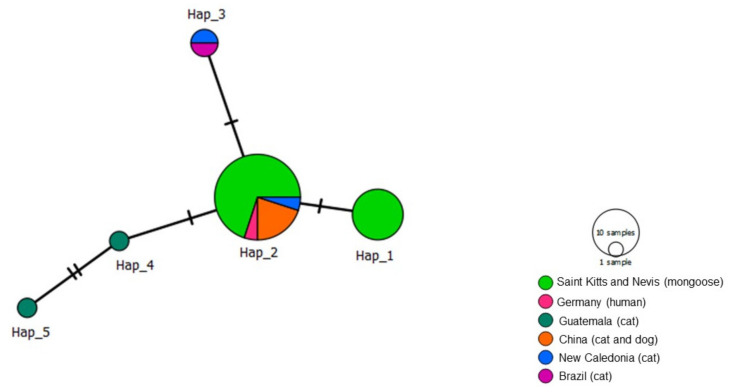
Haplotype network for *Bartonella henselae fstZ* sequences detected in mongooses and their fleas, Saint Kitts (haplotypes #1 and #2), combined with *Bartonella* sequences that were previously worldwide. Each small dash represents mutations. Dark circles represent median vectors. The haplotype network was generated with DNAsp data followed by analysis in PopArt using geographic coordinates and a TCS network.

Analysis of 31 *ftsZ* sequences including 21 from this study yielded five haplotypes (Table 4, Figure 10). Unlike the analyses for the other genes, and concurring with the phylogenetic analysis, our *ftsZ* sequences did not cluster into two clades. Rather, Haplotype #2 that contained *B. henselae* in mongooses from St. Kitts, as well as cats from Brazil, New Caledonia and China. Haplotype #1 arose from Haplotype #2 and consisted solely of *B. henselae* in mongooses from St. Kitts. Comparable sequences for *ftsZ* in *M. anakuma* were not available and thus not included in this analysis. 

Genetic diversity amongst the five genes was low, ranging from 0.544 to 0.655 (hd) (Table 4). Of the five genes analyzed, the highest diversity was observed in sequences for *Bartonella* targeting *nuoG*. 

## 4. Discussion

Bartonelloses are re-emerging infectious diseases with wildlife acting as major reservoirs for many of the zoonotic species. Traditionally viewed as self-limiting illnesses, these infections have now been implicated in a wide spectrum of human conditions, highlighting the importance of understanding the role of wildlife in the maintenance and spread of disease [3]. *Bartonella henselae* was previously described in mongooses from Japan [21] and Grenada [22] and in mongoose fleas from St. Kitts [26]. To the best of the authors’ knowledge, this is the first study investigating the molecular prevalence and genetic diversity of *Bartonella* in mongooses and their fleas from St. Kitts. 

The superorder Feliformia includes both domestic and big cats (lions, tigers, cheetahs), civets, hyaenas, and mongooses [12]. Among this group, *B. henselae* is the most prevalent *Bartonella* species [12]. This is supported by the present study, reinforcing the role of mongooses as potential reservoirs for *B. henselae*, as previously described in Japan [21] and Grenada [22]. The cat flea, *C. felis* is the main vector for *B. henselae* among cats [52], commonly infests both domestic cats and mongooses [53] and is believed to be key component in establishing *Bartonella* reservoirs in mongooses [21,22]. 

For each of the five loci, two haplotypes of *B. henselae* were detected in mongooses and *C. felis* fleas from this study. One haplotype was not previously described in other geographic locations, while the other was widely distributed and shared between mongooses, domestic and wild cats, cat fleas, and humans [22,35,54,55]. In fact, nBLAST analysis demonstrated the presence of one haplotype of *B. henselae* from St. Kitts to be 100.00% identical to *B. henselae* strain Houston-1 from a human in Germany [54], supported by phylogenetic and haplotype analyses. Such findings suggest that the Houston-1 strain circulates in the mongoose population of St. Kitts and this hypothesis is supported by previous studies conducted in Japan and Grenada, which demonstrated this strain in 16% and 100% of their positive mongooses, respectively [21,22]. Houston-1 is possibly more virulent to humans and have been implicated in most cat scratch disease cases [56]. Therefore, these findings warrant further investigation of circulating *B. henselae* strains in both mongooses and felines of St. Kitts by culturing and multi-locus sequence typing (MLST) in the future [57]. 

For *gltA,* two haplotypes of an undescribed *Bartonella* sp. were found circulating within the mongoose population of St. Kitts and for *rpoB*, *nuoG* and ITS, one haplotype of this species was observed. Previously detected in fleas from mongooses from the country [26], the sequences clustered with *Bartonella* sp. obtained from Japanese badgers (*M. anakuma)*. nBLAST analysis revealed *B. clarridgeiae* as the closest identified species to this *Bartonella* sp. (*gltA* fragments presenting 95.65–96.71% identity to *B. clarridgeiae*, KY91363). According to La Scola et al., *gltA* and *rpoB* have great discriminatory power and can be used for determining novel *Bartonella* species based on the percentage of identity, compared to recognized species available in GenBank [58]. *Bartonella* should be considered new species if a 327-bp *gltA* fragment shares < 96.0% or an 825-bp *rpoB* fragment shares < 95.4% similarity to validated species. In our study, a total of four *gltA* fragments met the criteria for determining a novel species (7F, 19S, 52F and 54F; accession numbers: MW728184, MW728185, MW238205 and MW728207). Likewise, *rpoB* sequences showed a low percentage of identity (95.95–6.71%) to *B. clarridgeiae*, however, fragments were shorter than the ones recommended by La Scola et al., precluding their use in this criterion.

Similar to the nBLAST results, phylogenetic analysis of four loci (*gltA*, *rpoB*, *nuoG*, ITS) revealed *B. clarridgeiae* as the closest identified species to this *Bartonella* sp. Even though the previous study of fleas from mongooses in St. Kitts [26] concluded that this unidentified species was most likely *B. henselae,* phylogenetic analyses based on four loci positioned the bacteria closest to *Bartonella* sp. detected in *M. anakuma*. Furthermore, the haplotype analysis (of four loci) demonstrated this unidentified species as being distinct from *B. henselae,* separated by many mutational events. The nBLAST findings, in addition to phylogenetic and haplotype analyzes of the four loci, support the classification of a novel species of *Bartonella* in mongooses and their *C. felis* fleas from St. Kitts, which we propose should be named ‘*Candidatus* Bartonella kittensis’. Bacterial culture must be performed in the future studies to fully characterize this novel species [59]. Regardless, implementation of the provisional status *Candidatus* for incompletely described procaryotes based on gene sequencing (prior to culture), is common, as seen with *Candidatus* Bartonella mayotimonensis’ and ‘*Candidatus* Bartonella merieuxii’ [60,61].

In addition to demonstrating two distinct *Bartonella* clades, this study also identified several samples that tested positive for both *B. henselae* and ‘*Candidatus* Bartonella kittensis’ when two or more sequences belonging to different genes were available. These results are likely due to co-infections with different *Bartonella* species, a phenomenon observed in rodents [62], domestic dogs [63], and cats [64,65]. Co-infections in wildlife carnivores are relatively uncommon, as opposed to the findings of this study [66,67,68]. This may also be the result of gene recombination, an important complication of *Bartonella* genotyping, and an evolutionary strategy for bacterial pathogenicity [57].

The present study reports the highest molecular occurrence (51.2%) of *Bartonella* recorded in mongooses worldwide. Differences between frequencies observed in St. Kitts and that of Japan (15.9%) [21] and Grenada (35.5%) [22] are likely due to the climate, vector, and mongoose distribution as well as the presence of a large feral cat population to act as a primary reservoir in St. Kitts [23]. More specifically, the relatively higher population density at the St. Kitts sampling site (4.0–7.8 mongoose/km^2^) [27] may favor intraspecies transmission of infectious diseases and caution should be taken when inferring these results island-wide. In addition to this, the use of a highly sensitive qPCR targeting *Bartonella nuoG* [33] may have detected animals with low bacterial loads, a common shortcoming of traditional diagnostic modalities [69]. The qPCR prevalence observed in pooled flea samples from mongooses (62.3%) was higher than previously described in individual fleas from St Kitts (10.3%) [26]. Direct comparisons between the two studies are difficult given the differences in methodology, namely the use of conventional versus quantitative PCR. Moreover, higher prevalence in the present study might be related to pooling flea samples. 

The mean number of *Bartonella* spp. *nuoG*-copies/µL in mongooses from St. Kitts was lower than that described in naturally infected cats from both southern Chile [70] and Brazil [33] using the same qPCR protocol. Direct comparisons with previous studies in mongooses was not possible due to a lack of published qPCR quantification data. This low number of *Bartonella* DNA copies is explained by its infection strategy, which results in chronic, asymptomatic, transient, and often undetectable infections in reservoirs species [7]. Absolute quantification of *Bartonella* spp. *nuoG*-copies was not possible in many of the tested samples due to discrepancies in Cq values. This may be a manifestation of the Monte Carlo effect, observed in samples with low initial DNA copies [34]. 

In this study, lower *Bartonella* DNA loads and lower occurrence were observed in the spleens when compared to blood. This could be explained by the dilution of spleen DNA samples prior to PCR amplification. However, similar findings were observed in mongooses from Grenada, where mixed tissues, including spleen, lymph node, and liver showed a lower *Bartonella* spp. prevalence (0.7%, 1/136) than blood samples (35.3%, 18/51) [22]. Differences in DNA loads between the spleen and blood may also reflect the role of the spleen in *Bartonella* infections, filtering and retaining infected erythrocytes, rather than acting as an infective niche [71]. Accordingly, splenectomized mice had 10-fold higher bacteremia than normal mice, highlighting the role of the spleen in clearing *Bartonella* infections [71]. Moreover, it is well established that *Bartonella* replicates within erythrocytes [72], possibly explaining higher bacterial loads measured in blood samples. Time of infection may also explain observed differences, with experimental studies showing higher *Bartonella birtlesii* recovery (CFU) from spleens in the early stages of infection compared to higher concentrations in blood seven-days post-infection [71].

The low haplotype diversity observed across all five genes is suggestive of high intraspecies similarities between the *Bartonella* sequences from the present study and those species described worldwide. Genomic variety between various strains of *B. henselae* is less than 1.00% and low haplotype diversity is an expected finding [73]. Findings from this study are comparable to that described in domestic cats from Chile, where a low haplotype diversity was described for *gltA* gene (hd = 0.601, π = 0.01) [70]. This contrasts with *gltA* analyses from bats (hd = 1.000, π = 0.104) and rodents (hd = 0.958, π = 0.024) from Brazil, in which the diversity of *Bartonella* is much higher [11,13].

*Bartonella* species adapt to specific environmental and ecological niches and co-evolve for optimal infection of a given vector and reservoir host [1]. Novel ‘*Candidatus* Bartonella kittensis’ may reflect a species seen only in mongooses and their associated cat fleas. The potential for this novel species to infect cats through the common vector, *C. felis* is yet to be determined, however, this species was not observed in previous molecular surveys of cats from St. Kitts [24]. Furthermore, the zoonotic potential for this novel species remains undetermined, and warrants further investigation in future studies. 

## 5. Conclusions

Lowly diverse *Bartonella* was prevalent in Small Indian mongooses and *C. felis* collected from them in St. Kitts. Mongooses and *C. felis* from St. Kitts are potential reservoirs for *B. henselae* and a novel species closely related to *Bartonella* sp. from Japanese badgers, proposed to be named ‘*Candidatus* Bartonella kittensis’. 

## Figures and Tables

**Table 1:** Summarized information on the different target genes, primer sets, amplification cycles, and product size used in conventional PCR assays in this study.

Target Locus	Primers (5′-3′)	Amplification Conditions			Size (bp)	Reference
*irbp* Mammalian species	F:TCCAACACCACCACTGAGATCTGGAC R:GTGAGGAAGAAATCGGACTGGCC	95 °C for 4 m 94 °C for 30 s 57 °C for 30 s 72 °C for 1 m 72 °C for 5 m	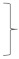	35 cycles	227	[31]
18S rRNA *Ctenocephalides felis*	F:TGCTCACCGTTTGACTTGG R:GTTTCTCAGGCTCCCTCTCC	95 °C for 3 m 94 °C for 30 s 60 °C for 45 s 72 °C for 1 m 72 °C for 7 m	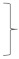	35 cycles	179	[32]
*gltA* *Bartonella* spp.	F:GCTATGTCTGCATTCTATCA R:GATCYTCAATCATTTCTTTCCA	95 °C for 2 m 94 °C for 30 s 52 °C for 30 s 72 °C for 1 m 72 °C for 5 m	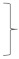	40 cycles	767	[36]
*rpoB* *Bartonella* spp.	F:CGATTYGCATCATCATTTTCC R:CGCATTATGGTCGTATTTGTCC	95 °C for 5 m 94 °C for 45 s 52 °C for 45 s 72 °C for 45 s 72 °C for 7 m	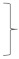	40 cycles	333	[37]
*ftsZ* *Bartonella* spp.	F:CATATGGTTTTCATTACTGCYGGTATGG R:TTCTTCGCGAATACGATTAGCAGCTTC	95 °C for 5 m 94 °C for 45 s 61 °C for 45 s 72 °C for 45 s 72 °C for 7 m	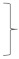	40 cycles	515	[37]
*nuoG* *Bartonella* spp.	F:GGCGTGATTGTTCTCGTTA R:CACGACCACGGCTATCAAT	94 °C for 5 m 94 °C for 30 s 53 °C for 30 s 72 °C for 30 s 72 °C for 7 m	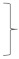	35 cycles	400	[38]
16-23S rRNA ITS *Bartonella* spp.	F:CTTCAGATGATGATCCCAAGCCTTYTGGCG R:GAACCGACGACCCCTGCTTGCAAAGCA	95 °C for 5 m 94 °C for 15 s 66 °C for 15 s 72 °C for 15 s 72 °C for 5 m	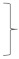	55 cycles	453–717	[39]

**Table 2:** Sequenced *Bartonella* spp. products from small Indian mongooses in Saint Kitts, with their closely BLASTn identity by target locus.

Locus	Occurrence	Identity by BLASTn	% Blast Identity	Accession Numbers
*gltA* (*n* = 42)	11/42	*B. henselae* from cats in Chile	97.80–100.00%	KY913625, KY913626, KY913627
	16/42	*B. henselae* from cat in Brazil	99.64–100.00%	MN107415
	2/42	*B. henselae* strain Houston-1 from human in Germany	99.87–100.00%	CP020742
	13/42	*Bartonella* sp. from *Meles Anakuma* in Japan	98.37–99.01%	CP019788
*rpoB* (*n* = 50)	16/50	*B. henselae* from a cat in Brazil	98.16–100.00%	MN107418
	6/50	*B. henselae* from a lion in South Africa	99.71–99.72%	KX499338
	28/50	*Bartonella* sp. from *Meles Anakuma* in Japan	99.71–99.72%	CP019788
*nuoG* (*n* = 21)	17/21	*B. henselae* strain Houston-1 from human in Germany	99.43–100.00%	CP020742
	4/21	*Bartonella* sp. from *Meles Anakuma* in Japan	96.74–96.78%	CP019788
ITS (*n* = 21)	29/31	*B. henselae* in a flea from Iran (KT314216), cheetah from Zimbabwe (KX499346) and cat from Brazil (MT095053)	99.58–100.00%	KT314216, KX499346, MT095053
	2/31	*Bartonella* sp. in a flea from St. Kitts	100.00%	MT048286
*fstZ* (*n* = 21)	21/21	*B. henselae* strain Houston-1 from human in Germany	99.81–100.00%	CP020742

**Table 3:** Distribution of *Bartonella* positive samples by sample type and species.

Sample	*B. henselae*	Unidentified *Bartonella* sp.	Co-Positive
Spleen (*n* = 16)	5 (31.2%)	9 (56.3%)	2 (12.5%)
Blood (*n* = 34)	21 (61.8%)	4 (11.8%)	9 (26.4%)
Fleas (*n* = 15)	7 (46.7%)	4 (26.7%)	4 (26.7%)
Overall (*n =* 65)	33 (50.8%)	17(26.2%)	15 (23.1%)

**Table 4:** Genetic diversity and polymorphisms of the *gltA*, *rpoB*, *fstZ*, *nuoG* and ITS sequences of Bartonella detected in Saint Kitts and worldwide.

Gene/Region	bp	N	VS	GC%	h	hd (Mean ± SD)	π (Mean ± SD)	K
*gltA*	568	50	70	36.8	6	0.547 ± 0.069	0.04864 ± 0.00670	27.62776
*rpoB*	318	77	40	38.0	4	0.595 ± 0.032	0.05454 ± 0.00325	17.34245
*fstZ*	491	31	5	44.9	5	0.544 ± 0.087	0.00151 ± 0.00040	0.73978
*nuoG*	336	26	51	40.2	5	0.655 ± 0.077	0.04744 ± 0.01376	15.93846
ITS	547	33	183	37.6	4	0.619 ± 0.049	0.08856 ± 0.03474	37.28409

N, number of sequences analysed; VS, number of variable sites; GC%, C + G content; h. number of haplotypes; hd, diversity of haplotypes; SD, standard deviation; π, nucleotide diversity (per site); K, nucleotide difference number.

## Data Availability

The data supporting the findings of this study are included within this article. Raw datasets generated during and/or analyzed during the current study are available from the corresponding author on reasonable request. Representative sequences were submitted to the GenBank database under the accession numbers: MW728178-MW728269, MW743240-MW743270, MW748304-MW748345, MW767381.

## Supplementary Materials

The following are available online at https://www.mdpi.com/article/10.3390/microorganisms9071350/s1, Table S1: The results obtained in quantitative real-time (q) PCR and conventional (c) PCR assays for Bartonella spp. for spleen, blood, and fleas from mongoose in Saint Kitts, Table S2–S4 Analysis of identity performed in BLAST for spleen, blood, and flea samples.

## References

[1] Chomel B.B., Boulouis H.J., Breitschwerdt E.B., Kasten R.W., Vayssier-Taussat M., Birtles R.J., Koehler J.E., Dehio C. (2009). Ecological Fitness and Strategies of Adaptation of *Bartonella* Species to Their Hosts and Vectors. Vet. Res..

[2] Chung C.Y., Kasten R.W., Paff S.M., Van Horn B.A., Vayssier-Taussat M., Boulouis H.J., Chomel B.B. (2004). *Bartonella* spp. DNA Associated with Biting Flies from California. Emerg. Infect. Dis..

[3] Breitschwerdt E. (2014). Bartonellosis: One Health Perspectives for an Emerging Infectious Disease. ILAR J..

[4] Bergh K., Bevanger L., Hanssen I., Løseth K. (2002). Low Prevalence of *Bartonella henselae* Infections in Norwegian Domestic and Feral Cats. APMIS.

[5] Chomel B.B., Carlos E.T., Kasten R.W., Yamamoto K., Chang C.C., Carlos R.S., Abenes M.V., Pajares C.M. (1999). *Bartonella henselae* and *Bartonella clarridgeiae* Infection in Domestic Cats from the Philippines. Am. J. Trop. Med. Hyg..

[6] Seubert A., Schulein R., Dehio C. (2001). Bacterial Persistence within Erythrocytes: A Unique Pathogenic Strategy of *Bartonella* spp.. Int. J. Med. Microbiol..

[7] Harms A., Dehio C. (2012). Intruders below the Radar: Molecular Pathogenesis of *Bartonella* spp.. Clin. Microbiol. Rev..

[8] Koehler J.E., Glaser C.A., Tappero J.W. (1994). Rochalimaea Henselae Infection: A New Zoonosis With the Domestic Cat as Reservoir. JAMA J. Am. Med. Assoc..

[9] Avidor B., Graidy M., Efrat G., Leibowitz C., Shapira G., Schattner A., Zimhony O., Giladi M. (2004). *Bartonella koehlerae*, a New Cat-Associated Agent of Culture-Negative Human Endocarditis. J. Clin. Microbiol..

[10] Roux V., Eykyn S.J., Wyllie S., Raoult D. (2000). *Bartonella vinsonii* subsp. Berkhoffii as an Agent of Afebrile Blood Culture-Negative Endocarditis in a Human. J. Clin. Microbiol..

[11] Gonçalves L.R., Favacho A.R.D.M., Roque A.L.R., Mendes N.S., Fidelis O.L., Benevenute J.L., Herrera H.M., D’Andrea P.S., de Lemos E.R.S., Machado R.Z. (2016). Association of *Bartonella* Species with Wild and Synanthropic Rodents in Different Brazilian Biomes. Appl. Environ. Microbiol..

[12] Kosoy M., Goodrich I. (2019). Comparative Ecology of Bartonella and Brucella Infections in Wild Carnivores. Front. Vet. Sci..

[13] André M.R., Gutiérrez R., Ikeda P., do Amaral R.B., de Sousa K.C.M., Nachum-Biala Y., Lima L., Teixeira M.M.G., Machado R.Z., Harrus S. (2019). Genetic Diversity of *Bartonella* spp. in Vampire Bats from Brazil. Transbound. Emerg. Dis..

[14] Nellis D.W., Everard C.O.R. (1983). The Biology of the Mongoose in the Caribbean. Stud. Fauna Curaçao Other Caribb. Islands.

[15] Louppe V., Leroy B., Herrel A., Veron G. (2020). The Globally Invasive Small Indian Mongoose Urva Auropunctata Is Likely to Spread with Climate Change. Sci. Rep..

[16] Everard C.O.R., Everard J.D. (1988). Mongoose Rabies. Rev. Infect. Dis..

[17] Shiokawa K., Llanes A., Hindoyan A., Cruz-Martinez L., Welcome S., Rajeev S. (2019). Peridomestic Small Indian Mongoose: An Invasive Species Posing as Potential Zoonotic Risk for Leptospirosis in the Caribbean. Acta Trop..

[18] Miller S., Zieger U., Ganser C., Satterlee S.A., Bankovich B., Amadi V., Hariharan H., Stone D., Wisely S.M. (2015). Influence of Land Use and Climate on Salmonella Carrier Status in the Small Indian Mongoose (*Herpestes auropunctatus*) in Grenada, West Indies. J. Wildl. Dis..

[19] Choudhary S., Zieger U., Sharma R.N., Chikweto A., Tiwari K.P., Ferreira L.R., Oliveira S., Barkley L.J., Verma S.K., Kwok O.C.H. (2013). Isolation and Rflp Genotyping of Toxoplasma Gondii From the Mongoose (*Herpestes auropunctatus*) in Grenada, West Indies. J. Zoo Wildl. Med..

[20] Rhynd K.J.R., Leighton P.A., Elcock D.A., Whitehall P.J., Rycroft A., Macgregor S.K. (2014). Prevalence of Salmonella spp. and Thermophilic Campylobacter spp. in the Small Asian Mongoose (*Herpestes javanicus*) in Barbados, West Indies. J. Zoo Wildl. Med..

[21] Sato S., Kabeya H., Shigematsu Y., Sentsui H., Une Y., Minami M., Murata K., Ogura G., Maruyama S. (2013). Small Indian Mongooses and Masked Palm Civets Serve as New Reservoirs of *Bartonella henselae* and Potential Sources of Infection for Humans. Clin. Microbiol. Infect..

[22] Jaffe D.A., Chomel B.B., Kasten R.W., Breitschwerdt E.B., Maggi R.G., McLeish A., Zieger U. (2018). *Bartonella henselae* in Small Indian Mongooses (*Herpestes auropunctatus*) from Grenada, West Indies. Vet. Microbiol..

[23] Kelly P.J., Moura L., Miller T., Thurk J., Perreault N., Weil A., Maggio R., Lucas H., Breitschwerdt E. (2010). Feline Immunodeficiency Virus, Feline Leukemia Virus and *Bartonella* Species in Stray Cats on St Kitts, West Indies. J. Feline Med. Surg..

[24] Huang K., Kelly P.J., Zhang J., Yang Y., Liu W., Kalalah A., Wang C. (2019). Molecular Detection of *Bartonella* spp. In China and St. Kitts. Can. J. Infect. Dis. Med. Microbiol..

[25] Reeves W.K., Beck J., Orlova M.V., Daly J.L., Pippin K., Revan F., Loftis A.D. (2016). Ecology of Bats, Their Ectoparasites, and Associated Pathogens on Saint Kitts Island. J. Med. Entomol..

[26] Fang K., Philpot K., Chi X., Ketzis J., Du A., Yao C. (2021). Small Indian Mongooses (*Herpestes auropunctatus*) Serve As Reservoirs of *Bartonella henselae* and Rickettsia felis Vectored by Ctenocephalides felis. Vector Borne Zoonotic Dis..

[27] Sauvé C., Berentsen A., Conan A., Criuz-Martinez L., Gilbert A., Leighton P. (2021). Habitat-Specific Mongoose Density Estimates and Factors Affecting Traping Success, a Field Study in St. Kitts, West Indies. Prep.

[28] Leary S., Underwood W., Anthony R., Cartner S., Corey D., Grandin T., Gwaltney-Brant S., McCrackin M.A., Greenacre C., Meyer R. (2013). AVMA Guidelines for the Euthanasia of Animals: 2013 Edition. Sci. World.

[29] Linardi P.M., Santos J.L.C. (2012). Ctenocephalides Felis Felis vs. Ctenocephalides Canis (Siphonaptera: Pulicidae): Some Issues in Correctly Identify These Species. Rev. Bras. Parasitol. Vet..

[30] Jansson L., Hedman J. (2019). Challenging the Proposed Causes of the PCR Plateau Phase. Biomol. Detect. Quantif..

[31] Ferreira E.C., Gontijo C.M., Cruz I., Melo M.N., Silva A.M. (2010). Alternative PCR Protocol Using a Single Primer Set for Assessing DNA Quality in Several Tissues from a Large Variety of Mammalian Species Living in Areas Endemic for Leishmaniasis. Mem. Inst. Oswaldo Cruz.

[32] Reif K.E., Stout R.W., Henry G.C., Foil L.D., Macaluso K.R. (2008). Prevalence and Infection Load Dynamics of Rickettsia Felis in Actively Feeding Cat Fleas. PLoS ONE.

[33] André M.R., Dumler J.S., Herrera H.M., Gonçalves L.R., de Sousa K.C.M., Scorpio D.G., de Santis A.C.G.A., Domingos I.H., de Macedo G.C., Machado R.Z. (2015). Assessment of a Quantitative 5’ Nuclease Real-Time Polymerase Chain Reaction Using the Nicotinamide Adenine Dinucleotide Dehydrogenase Gamma Subunit (NuoG) for *Bartonella* Species in Domiciled and Stray Cats in Brazil. J. Feline Med. Surg..

[34] Bustin S.A., Benes V., Garson J.A., Hellemans J., Huggett J., Kubista M., Mueller R., Nolan T., Pfaffl M.W., Shipley G.L. (2009). The MIQE Guidelines: Minimum Information for Publication of Quantitative Real-Time PCR Experiments. Clin. Chem..

[35] Müller A., Rodríguez E., Walker R., Bittencourt P., Pérez-Macchi S., Gonçalves L.R., Machado R.Z., André M.R. (2018). Occurrence and Genetic Diversity of *Bartonella* spp. (Rhizobiales: Bartonellaceae) and Rickettsia spp. (Rickettsiales: Rickettsiaceae) in Cat Fleas (Siphonaptera: Pulicidae) From Chile. J. Med. Entomol..

[36] Billeter S.A., Gundi V.A.K.B., Rood M.P., Kosoy M.Y. (2011). Molecular Detection and Identification of *Bartonella* Species in Xenopsylla Cheopis Fleas (Siphonaptera: Pulicidae) Collected from Rattus Norvegicus Rats in Los Angeles, California. Appl. Environ. Microbiol..

[37] Paziewska A., Harris P.D., Zwolińska L., Bajer A., Siński E. (2011). Recombination Within and Between Species of the Alpha Proteobacterium *Bartonella* Infecting Rodents. Microb. Ecol..

[38] Colborn J.M., Kosoy M.Y., Motin V.L., Telepnev M.V., Valbuena G., Myint K.S., Fofanov Y., Putonti C., Feng C., Peruski L. (2010). Improved Detection of *Bartonella* DNA in Mammalian Hosts and Arthropod Vectors by Real-Time PCR Using the NADH Dehydrogenase Gamma Subunit (NuoG). J. Clin. Microbiol..

[39] Dillon B., Iredell J., Breitschwerdt E.B., Maggi R.G. (2005). Potential Limitations of the 16S-23S RRNA Intergenic Region for Molecular Detection of *Bartonella* Species [5] (Multiple Letters). J. Clin. Microbiol..

[40] Ewing B., Green P. (1998). Base-Calling of Automated Sequencer Traces Using Phred. II. Error Probabilities. Genome Res..

[41] Ewing B., Hillier L.D., Wendl M.C., Green P. (1998). Base-Calling of Automated Sequencer Traces Using Phred. I. Accuracy Assessment. Genome Res..

[42] Altschul S.F., Gish W., Miller W., Myers E.W., Lipman D.J. (1990). Basic Local Alignment Search Tool. J. Mol. Biol..

[43] Katoh K., Standley D.M. (2013). MAFFT Multiple Sequence Alignment Software Version 7: Improvements in Performance and Usability. Mol. Biol. Evol..

[44] Darriba D., Taboada G.L., Doallo R., Posada D. (2014). High-Performance Computing Selection of Models of DNA Substitution for Multicore Clusters. Int. J. High Perform. Comput. Appl..

[45] Miller M.A., Pfeiffer W., Schwartz T. Creating the CIPRES Science Gateway for Inference of Large Phylogenetic Trees. Proceedings of the 2010 Gateway Computing Environments Workshop, GCE 2010.

[46] Posada D., Buckley T.R. (2004). Model Selection and Model Averaging in Phylogenetics: Advantages of Akaike Information Criterion and Bayesian Approaches over Likelihood Ratio Tests. Syst. Biol..

[47] Ronquist F., Huelsenbeck J.P. (2003). MrBayes 3: Bayesian Phylogenetic Inference under Mixed Models. Bioinformatics.

[48] Stöver B.C., Müller K.F. (2010). TreeGraph 2: Combining and Visualizing Evidence from Different Phylogenetic Analyses. BMC Bioinform..

[49] Librado P., Rozas J. (2009). DnaSP v5: A Software for Comprehensive Analysis of DNA Polymorphism Data. Bioinformatics.

[50] Clement M., Posada D., Crandall K.A. (2000). TCS: A Computer Program to Estimate Gene Genealogies. Mol. Ecol..

[51] Huson D.H., Bryant D. (2005). Application of Phylogenetic Networks in Evolutionary Studies. Mol. Biol. Evol..

[52] Chomel B.B., Kasten R.W., Floyd-Hawkins K., Chi B., Yamamoto K., Roberts-Wilson J., Gurfield A.N., Abbott R.C., Pedersen N.C., Koehler J.E. (1996). Experimental Transmission of *Bartonella henselae* by the Cat Flea. J. Clin. Microbiol..

[53] Cheng T., Halper B., Siebert J., Cruz-Martinez L., Chapwanya A., Kelly P., Ketzis J.K., Vessell J., Köster L., Yao C. (2018). Parasites of Small Indian Mongoose, Herpestes Auropunctatus, on St. Kitts, West Indies. Parasitol. Res..

[54] Omasits U., Varadarajan A.R., Schmid M., Goetze S., Melidis D., Bourqui M., Nikolayeva O., Québatte M., Patrignani A., Dehio C. (2017). An Integrative Strategy to Identify the Entire Protein Coding Potential of Prokaryotic Genomes by Proteogenomics. Genome Res..

[55] Pedrassani D., Biolchi J., Gonçalves L.R., Mendes N.S., Zanatto D.C.D.S., Calchi A.C., Machado R.Z., André M.R. (2019). Molecular Detection of Vector-Borne Agents in Cats in Southern Brazil. Rev. Bras. Parasitol. Vet..

[56] Boulouis H.J., Chang C.C., Henn J.B., Kasten R.W., Chomel B.B. (2005). Factors Associated with the Rapid Emergence of Zoonotic Bartonella Infections. Vet. Res..

[57] Kosoy M., McKee C., Albayrak L., Fofanov Y. (2018). Genotyping of Bartonella Bacteria and Their Animal Hosts: Current Status and Perspectives. Parasitology.

[58] La Scola B., Zeaiter Z., Khamis A., Raoult D. (2003). Gene-Sequence-Based Criteria for Species Definition in Bacteriology: The Bartonella Paradigm. Trends Microbiol..

[59] Gutiérrez R., Vayssier-Taussat M., Buffet J.P., Harrus S. (2017). Guidelines for the Isolation, Molecular Detection, and Characterization of Bartonella Species. Vector Borne Zoonotic Dis..

[60] Lin E.Y., Tsigrelis C., Baddour L.M., Lepidi H., Rolain J.M., Patel R., Raoult D. (2010). Candidatus Bartonella Mayotimonensis and Endocarditis. Emerg. Infect. Dis..

[61] Chomel B.B., McMillan-Cole A.C., Kasten R.W., Stuckey M.J., Sato S., Maruyama S., Diniz P.P.V.P., Breitschwerdt E.B. (2012). Candidatus Bartonella Merieuxii, a Potential New Zoonotic *Bartonella* Species in Canids from Iraq. PLoS Negl. Trop. Dis..

[62] Gutiérrez R., Morick D., Cohen C., Hawlena H., Harrus S. (2014). The Effect of Ecological and Temporal Factors on the Composition of *Bartonella* Infection in Rodents and Their Fleas. ISME J..

[63] Pérez C., Maggi R.G., Diniz P.P.V.P., Breitschwerdt E.B. (2011). Molecular and Serological Diagnosis of *Bartonella* Infection in 61 Dogs from the United States. J. Vet. Intern. Med..

[64] Gutiérrez R., Morick D., Gross I., Winkler R., Abdeen Z., Harrus S. (2013). Bartonellae in Domestic and Stray Cats from Israel: Comparison of Bacterial Cultures and High-Resolution Melt Real-Time PCR as Diagnostic Methods. Vector Borne Zoonotic Dis..

[65] Gurfield A.N., Boulouis H.J., Chomel B.B., Heller R., Kasten R.W., Yamamoto K., Piemont Y. (1997). Coinfection with *Bartonella clarridgeiae* and *Bartonella henselae* and with Different *Bartonella henselae* Strains in Domestic Cats. J. Clin. Microbiol..

[66] Bai Y., Gilbert A., Fox K., Osikowicz L., Kosoy M. (2016). *Bartonella rochalimae* and B. Vinsonii Subsp. Berkhoffii in Wild Carnivores from Colorado, USA. J. Wildl. Dis..

[67] Gerrikagoitia X., Gil H., García-Esteban C., Anda P., Juste R.A., Barral M. (2012). Presence of *Bartonella* Species in Wild Carnivores of Northern Spain. Appl. Environ. Microbiol..

[68] López-Pérez A.M., Osikowicz L., Bai Y., Montenieri J., Rubio A., Moreno K., Gage K., Suzán G., Kosoy M. (2017). Prevalence and Phylogenetic Analysis of *Bartonella* Species of Wild Carnivores and Their Fleas in Northwestern Mexico. Ecohealth.

[69] Agan B.K., Dolan M.J. (2002). Laboratory Diagnosis of *Bartonella* Infections. Clin. Lab. Med..

[70] Müller A., Walker R., Bittencourt P., MacHado R.Z., Benevenute J.L., Do Amaral R.B., Gonçalves L.R., André M.R. (2017). Prevalence, Hematological Findings and Genetic Diversity of *Bartonella* spp. in Domestic Cats from Valdivia, Southern Chile. Parasitology.

[71] Deng H.K., Le Rhun D., Lecuelle B., Le Naour E., Vayssier-Taussat M. (2012). Role of the Spleen in *Bartonella* spp. Infection. FEMS Immunol. Med. Microbiol..

[72] Schülein R., Seubert A., Gille C., Lanz C., Hansmann Y., Piémont Y., Dehio C. (2001). Invasion and Persistent Intracellular Colonization of Erythrocytes: A Unique Parasitic Strategy of the Emerging Pathogen *Bartonella*. J. Exp. Med..

[73] Guy L., Nystedt B., Toft C., Zaremba-Niedzwiedzka K., Berglund E.C., Granberg F., Näslund K., Eriksson A.S., Andersson S.G.E. (2013). A Gene Transfer Agent and a Dynamic Repertoire of Secretion Systems Hold the Keys to the Explosive Radiation of the Emerging Pathogen *Bartonella*. PLoS Genet..

