# Supplementary material for: Molecular Survey and Genetic Diversity of Bartonella spp. in Small Indian Mongooses (Urva auropunctata) and Their Fleas on Saint Kitts, West Indies
Source: Microorganisms. 2021 Jun 22;9(7):1350. doi: 10.3390/microorganisms9071350 (PMC8306750; doi:10.3390/microorganisms9071350)
Supplement: Supplementary file 1 [file microorganisms-09-01350-s001.zip › microorganisms-1244055-supplementary.pdf]

Supplemental Tables

Table S1: The results obtained in quantitative real-time (q)PCR and conventional (c)PCR assays for *Bartonella* spp. for spleen, blood and fleas from mongoose in Saint Kitts.

	***Bartonella* spp. *nouG*-qPCR**		***Bartonella* spp. cPCR**				
**Sample ID**	**Cq Mean**	**Quantification Mean (copies/uL)**	***gltA***	***rpoB***	***fstZ***	***nuoG***	**ITS**
Spleen							
**3S**	31.16	5.28×10^{-1}	+S	+S	+S	+S	+S
**10S**	N/A	N/A	+NS	+NS	-	+NS	+NS
**19S**	N/A	N/A	+S	+S	-	-	-
**24S**	N/A	N/A	+S	+NS	+S	+S	+S
**26S**	N/A	N/A	+NS	+S	-	-	-
**30S**	32.61	4.74×10^{-2}	+S	+S	+NS	+NS	+S
**32S**	N/A	N/A	+NS	+S	-	+NS	-
**33S**	N/A	N/A	+NS	+S	-	+NS	-
**34S**	N/A	N/A	+NS	+NS	-	+NS	+NS
**38S**	29.92	1.15	+S	+S	+S	+S	+S
**41S**	N/A	N/A	+NS	+S	-	+NS	-
**44S**	N/A	N/A	+S	+S	+S	+S	+S
**45S**	N/A	N/A	-	+S	-	-	-
**49S**	32.08	2.58×10^{-1}	+S	+S	+S	+S	+S
**53S**	N/A	N/A	+S	+S	+S	+S	+S
**55S**	N/A	N/A	+NS	+S	-	-	+NS
**56S**	N/A	N/A	+NS	+S	-	-	-
**62S**	N/A	N/A	-	+NS	-	-	-
Blood							
**1B**	30.28	1.71×10^{1}	+NS	+S	+S	+NS	+NS
**3B**	30.43	1.56×10^{1}	+NS	+S	+NS	+NS	+NS
**4B**	29.53	2.29×10^{1}	+S	+S	+S	+S	+S
**5B**	31.65	1.62×10^{0}	+NS	+NS	+NS	+NS	+NS
**8B**	N/A	N/A	+NS	+NS	+NS	-	+NS
**11B**	30.77	2.90×10^{1}	+NS	+NS	+S	+S	+S
**12B**	29.97	2.11×10^{1}	+S	+NS	+S	+S	+S
**13B**	31.6	7.22×10^{0}	-	-	-	-	-
**16B**	36.66	4.97×10^{-1}	-	-	-	-	-
**18B**	N/A	N/A	+NS	-	-	-	-
**19B**	N/A	N/A	-	-	-	-	-
**20B**	33	2.89×10^{0}	-	-	-	-	-
**21B**	30.82	2.81×10^{1}	+S	+NS	+NS	+S	+S
**22B**	33.1	2.72×10^{0}	+S	+NS	-	+NS	+S
**23B**	N/A	N/A	+NS	+NS	-	-	-
**24B**	29.4	3.06×10^{1}	+S	+NS	+NS	+NS	+NS
**25B**	36.72	5.88×10^{-1}	+NS	+NS	-	-	+NS

**26B**	N/A	N/A	+NS	+S	-	-	-
**27B**	N/A	N/A	+NS	+S	+NS	+NS	+NS
**31B**	N/A	N/A	+S	+S	+NS	+NS	+NS
**32B**	29.4	7.08×10^{1}	+S	+NS	+S	+S	+S
**36B**	N/A	N/A	+NS	+NS	-	+NS	+NS
**37B**	35.99	4.15×10^{-1}	+S	+NS	+NS	+NS	+NS
**38B**	31.51	1.41×10^{1}	+S	+NS	+NS	+NS	+NS
**42B**	N/A	N/A	+S	+NS	+NS	+NS	+NS
**43B**	N/A	N/A	+S	+S	+S	+S	+S
**44B**	30.8	2.25×10^{1}	+S	+S	+NS	+NS	+NS
**46B**	30.36	2.99×10^{1}	+NS	+S	+NS	+NS	+S
**47B**	29.21	6.37×10^{1}	+S	+S	+NS	+NS	+NS
**48B**	N/A	N/A	+S	+S	-	+S	+S
**49B**	30.86	2.20×10^{1}	+S	+S	+S	+S	+S
**50B**	32.55	7.18×10^{0}	+S	+S	+NS	+NS	+S
**51B**	N/A	N/A	+NS	+NS	+NS	+NS	+NS
**52B**	N/A	N/A	+NS	+NS	+NS	+NS	+NS
**53B**	N/A	N/A	+NS	+S	+NS	-	+S
**57B**	31.53	1.75×10^{1}	+NS	+S	-	+NS	-
**58B**	31.3	2.04×10^{1}	+S	+S	-	+NS	-
**59B**	32.05	1.25×10^{1}	+S	+NS	+NS	+NS	+NS
**60B**	32.2	9.73×10^{0}	+S	+NS	+NS	+NS	+NS
**61B**	29.16	6.52×10^{1}	+S	+S	+NS	+NS	+NS
**64B**	34.04	2.74×10^{0}	+NS	+NS	+NS	+NS	-
**65B**	31.7	1.25×10^{1}	+S	+S	+S	+S	+S
**67B**	31.36	1.58×10^{1}	+NS	+S	+S	+NS	+S
**69B**	31.4	1.52×10^{1}	+S	+S	+NS	+S	+S
**70B**	27.5	1.92×10^{2}	+S	+S	+NS	+NS	+NS
**71B**	31.57	1.36×10^{1}	+NS	+S	+S	+S	+S
Flea							
**7F**	23.51	1.60×10^{3}	+S	+S	+S	+S	+S
**8F**	33.3	1.97×10^{0}	+NS	+NS	+NS	+NS	-
**9F**	32.97	2.67×10^{0}	+NS	+NS	+NS	+NS	+NS
**12F**	N/A	N/A	+NS	+NS	-	+NS	+S
**13F**	28.86	4.67×10^{1}	+NS	+NS	+NS	+NS	+NS
**16F**	25.67	3.85×10^{2}	+NS	+S	+S	+NS	+NS
**20F**	23.2	1.98×10^{3}	+S	+S	+S	+S	+S
**21F**	N/A	N/A	+NS	-	-	+NS	+NS
**22F**	N/A	N/A	+NS	+NS	+NS	+NS	-
**24F**	21.96	4.47×10^{3}	+S	+NS	+NS	+NS	+NS
**25F**	N/A	N/A	+NS	-	-	+NS	+NS
**27F**	N/A	N/A	+NS	+NS	-	+NS	-
**28F**	33.29	2.51×10^{0}	+NS	+NS	+NS	+NS	+NS

**30F**	N/A	N/A	+NS	+NS	+NS	+NS	-
**31F**	N/A	N/A	-	+NS	-	+NS	-
**33F**	N/A	N/A	+NS	+NS	+NS	+NS	+NS
**35F**	30.17	1.98×10^{1}	+NS	+NS	+NS	+NS	-
**37F**	27.53	1.13×10^{2}	+NS	+S	+NS	+NS	+NS
**38F**	23.08	2.73×10^{-1}	+S	+S	+S	+S	+S
**39F**	36.64	2.73×10^{-1}	+NS	+NS	+NS	+NS	+NS
**41F**	N/A	N/A	+NS	+NS	+NS	+NS	+NS
**42F**	27.93	8.65×10^{1}	+NS	+S	-	+NS	-
**43F**	32.53	4.15×10^{0}	+NS	+NS	-	+NS	-
**44F**	N/A	N/A	-	+NS	-	+NS	-
**48F**	N/A	N/A	+NS	+NS	+NS	+NS	+NS
**50F**	25.54	4.19×10^{2}	+S	+NS	+S	+S	+S
**51F**	26.59	6.03×10^{2}	+S	+S	N	+S	+S
**52F**	23.17	5.51×10^{3}	+S	+S	N	+S	+S
**54F**	26.46	6.57×10^{2}	+S	+S	N	+S	+S
**59F**	22.88	6.66×10^{3}	-	+S	+NS	+NS	+NS
**88F**	N/A	N/A	-	+NS	+NS	+NS	+NS
**103F**	N/A	N/A	-	+NS	-	+NS	-
**112F**	33.18	9.85×10^{0}	-	+NS	-	+NS	-

Note: ID, Identification; - , negative sample; +S, qPCR positive samples that were sequenced; +NS, cPCR positive samples that were not sequenced or were sequenced and yielded poor quality electropherograms.

Table S2: Analysis of identity performed in BLAST for Spleen samples

Sample	Gene/ Locus	Query Cover	Identity %	Identity/host/country/GenBank accession number	
3	*gltA*	100.00%	100.00%	*Bartonella henselae* in cat from Brazil	MN107415
	ITS	100.00%	100.00%	*Bartonella henselae* in cheetah (*Acinonyx jubatus)* from Zimbabwe	KX499346
	nuoG	100.00%	99.71%	*Bartonella henselae* in human from Germany	CP020742
	rpoB	100.00%	99.72%	*Bartonella henselae* in cat from Brazil	MN107418
19	*gltA*	100.00%	98.79%	*Bartonella* sp. in *Meles anakuma* from Japan	CP019788
	rpoB	100.00%	98.08%	*Bartonella* sp. in *Meles anakuma* from Japan	CP019788
24	*fstZ*	100.00%	99.81%	*Bartonella henselae* in human from Germany	CP020742
	gltA	100.00%	100.00%	*Bartonella henselae* in fleas removed from cats in Chile	KY913626
	ITS	100.00%	100.00%	*Bartonella henselae* in cat from Brazil	MT095053
	nuoG	100.00%	99.43%	*Bartonella henselae* in human from Germany	CP020742
26	*rpoB*	100.00%	98.01%	*Bartonella* sp. in *Meles anakuma* from Japan	CP019788
30*	*fstZ*	100.00%	100.00%	*Bartonella henselae* in human from Germany	CP020742
	gltA	99.00%	99.87%	*Bartonella henselae* in human from Germany	CP020742
	ITS	100.00%	100.00%	*Bartonella henselae* in cat from Brazil	MT095053
	rpoB	100.00%	98.07%	*Bartonella* sp. in *Meles anakuma* from Japan	CP019788
32	*rpoB*	100.00%	97.99%	*Bartonella* sp. in *Meles anakuma* from Japan	CP019788

33	*rpoB*	100.00%	98.01%	*Bartonella* sp. in *Meles anakuma* from Japan	CP019788
38	*fstZ*	100.00%	99.81%	*Bartonella henselae* in human from Germany	CP020742
	gltA	100.00%	100.00%	*Bartonella henselae* in fleas removed from cats in Chile	KY913627
	ITS	100.00%	100.00%	*Bartonella henselae* in cat from Brazil	MT095053
	nuoG	100.00%	99.71%	*Bartonella henselae* in human from Germany	CP020742
	rpoB	100.00%	99.71%	*Bartonella henselae* in cat from Brazil	CP019788
41	*rpoB*	100.00%	98.02%	*Bartonella* sp. in *Meles anakuma* from Japan	CP019788
44	*fstZ*	100.00%	100.00%	*Bartonella henselae* in human from Germany	CP020742
	gltA	100.00%	100.00%	*Bartonella henselae* in cat from Brazil	MN107415
	ITS	100.00%	100.00%	*Bartonella henselae* in cheetah (*Acinonyx jubatus)* from Zimbabwe	KX499346
	nuoG	100.00%	99.70%	*Bartonella henselae* in human from Germany	CP020742
	rpoB	100.00%	99.71%	*Bartonella henselae* in lion (*Panthera leo*) from South Africa	KX499338
45	*rpoB*	100.00%	98.03%	*Bartonella* sp. in *Meles anakuma* from Japan	CP019788
48	*rpoB*	100.00%	98.03%	*Bartonella* sp. in *Meles anakuma* from Japan	CP019788
49*	*fstZ*	100.00%	100.00%	*Bartonella henselae* in human from Germany	CP020742
	gltA	100.00%	100.00%	*Bartonella henselae* in fleas removed from cats in Chile	KY913626
	ITS	100.00%	100.00%	*Bartonella henselae* in cheetah (*Acinonyx jubatus)* from Zimbabwe	KX499346
	rpoB	100.00%	98.02%	*Bartonella* sp. in *Meles anakuma* from Japan	CP019788
53	*fstZ*	100.00%	100.00%	*Bartonella henselae* in human from Germany	CP020742
	ITS	100.00%	99.58%	*Bartonella henselae* in cat from Brazil	MT095053
	nuoG	100.00%	100.00%	*Bartonella henselae* in human from Germany	CP020742
	rpoB	100.00%	99.72%	*Bartonella henselae* in lion (*Panthera leo*) from South Africa	KX499338
55	*rpoB*	100.00%	98.02%	*Bartonella* sp. in *Meles anakuma* from Japan	CP019788
56	*rpoB*	100.00%	98.01%	*Bartonella* sp. in *Meles anakuma* from Japan	CP019788

***Co positive sample**

Table S3: Analysis of identity performed in BLAST for blood samples

Sample	Gene/ Locus	Query Cover	Identity %	Identity/host/country/GenBank accession number	
**1***	*fstZ*	100.00%	100.00%	*Bartonella henselae* in human from Germany	CP020742
	rpoB	100.00%	98.00%	*Bartonella* sp. in *Meles anakuma* from Japan	CP019788
**3**	*fstZ*	100.00%	100.00%	*Bartonella henselae* in human from Germany	CP020742
	rpoB	100.00%	99.71%	*Bartonella henselae* in cat from Brazil	MN107418
**4**	*fstz*	100.00%	99.81%	*Bartonella henselae* in human from Germany	CP020742
	gltA	100.00%	100%	*Bartonella henselae* in cat from Brazil	MN107415
	ITS	100.00%	99.82%	*Bartonella henselae* in cat from Brazil	MT095053
	nuoG	100.00%	99.43%	*Bartonella henselae* in human from Germany	CP020742
	rpoB	100.00%	99.72%	*Bartonella henselae* in cat from Brazil	MN107418
**11**	*fstZ*	100.00%	100.00%	*Bartonella henselae* in human from Germany	CP020742
	ITS	100.00%	100.00%	*Bartonella henselae* in cheetah (*Acinonyx jubatus)* from Zimbabwe	KX499346
	nuoG	100.00%	99.70%	*Bartonella henselae* in human from Germany	CP020742
**12**	*fstZ*	100.00%	100.00%	*Bartonella henselae* in human from Germany	CP020742
	gltA	100.00%	100.00%	*Bartonella henselae* in fleas removed from cats in Chile	KY913626

	ITS	100.00%	100.00%	*Bartonella henselae* in cheetah (*Acinonyx jubatus)* from Zimbabwe	KX499346
	nuoG	100.00%	99.71%	*Bartonella henselae* in human from Germany	CP020742
**21***	*gltA*	100.00%	98.65%	*Bartonella* sp. in *Meles anakuma* from Japan	CP019788
	nuoG	100.00%	99.70%	*Bartonella henselae* in human from Germany	CP020742
**22***	*gltA*	100.00%	98.82%	*Bartonella* sp. in *Meles anakuma* from Japan	CP019788
	ITS	100.00%	100.00%	*Bartonella henselae* in cat from Brazil	MT095053
**24**	*gltA*	100.00%	100.00%	*Bartonella henselae* in cat from Brazil	MN107415
**26**	*rpoB*	100.00%	98.02%	*Bartonella* sp. in *Meles anakuma* from Japan	CP019788
**27**	*rpoB*	100.00%	99.72%	*Bartonella henselae* in cat from Brazil	MN107418
**31**	*gltA*	100.00%	100.00%	*Bartonella henselae* in fleas removed from cats in Chile	KY913626
	rpoB	100.00%	99.72%	*Bartonella henselae* in cat from Brazil	MN107418
**32**	*gltA*	100.00%	100.00%	*Bartonella henselae* in fleas removed from cats in Chile	KY913626
	ITS	100.00%	100.00%	*Bartonella henselae* in cat from Brazil	MT095053
	nuoG	100.00%	99.70%	*Bartonella henselae* in human from Germany	CP020742
**37**	*gltA*	100.00%	99.86%	*Bartonella henselae* in fleas removed from cats in Chile	KY913625
**38**	*gltA*	100.00%	100.00%	*Bartonella henselae* in cat from Brazil	MN107415
**42**	*gltA*	100.00%	98.73%	*Bartonella* sp. in *Meles anakuma* from Japan	CP019788
**43**	*fstZ*	100.00%	100.00%	*Bartonella henselae* in human from Germany	CP020742
	gltA	100.00%	100.00%	*Bartonella henselae* in cat from Brazil	MN107415
	ITS	100.00%	100.00%	*Bartonella henselae* in cheetah (*Acinonyx jubatus)* from Zimbabwe	KX499346
	nuoG	100.00%	99.70%	*Bartonella henselae* in human from Germany	CP020742
	rpoB	100.00%	99.72%	*Bartonella henselae* in lion (*Panthera leo*) from South Africa	KX499338
**44**	*gltA*	100.00%	100.00%	*Bartonella henselae* in cat from Brazil	MN107415
	rpoB	100.00%	99.72%	*Bartonella henselae* in cat from Brazil	MN107418
**46***	*gltA*	100.00%	98.82%	*Bartonella* sp. in *Meles anakuma* from Japan	CP019788
	ITS	100.00%	100.00%	*Bartonella henselae* in cat from Brazil	MT095053
	rpoB	100.00%	98.02%	*Bartonella* sp. in *Meles anakuma* from Japan	CP019788
**47**	*gltA*	100.00%	100.00%	*Bartonella henselae* in cat from Brazil	MN107415
	rpoB	100.00%	99.72%	*Bartonella henselae* in cat from Brazil	MN107418
**48***	*gltA*	100.00%	99.01%	*Bartonella* sp. in *Meles anakuma* from Japan	CP019788
	ITS	100.00%	99.82%	*Bartonella henselae* in flea from Iran	KT314216
	nuoG	100.00%	96.74%	*Bartonella* sp. in *Meles anakuma* from Japan	CP019788
	rpoB	100.00%	98.03%	*Bartonella* sp. in *Meles anakuma* from Japan	CP019788
**49**	*fstZ*	100.00%	100.00%	*Bartonella henselae* in human from Germany	CP020742
	gltA	100.00%	100.00%	*Bartonella henselae* in cat from Brazil	MN107415
	ITS	100.00%	100.00%	*Bartonella henselae* in cheetah (*Acinonyx jubatus)* from Zimbabwe	KX499346
	nuoG	100.00%	99.71%	*Bartonella henselae* in human from Germany	CP020742
	rpoB	100.00%	99.72%	*Bartonella henselae* in lion (*Panthera leo*) from South Africa	KX499338
**50***	*gltA*	100.00%	98.82%	*Bartonella* sp. in *Meles anakuma* from Japan	CP019788
	ITS	100.00%	100.00%	*Bartonella henselae* in flea from Iran	KT314216
	rpoB	100.00%	98.07%	*Bartonella* sp. in *Meles anakuma* from Japan	CP019788
**53**	ITS	100.00%	100.00%	*Bartonella henselae* in cat from Brazil	MT095053
	rpoB	100.00%	98.08%	*Bartonella* sp. in *Meles anakuma* from Japan	CP019788

**57**	*rpoB*	100.00%	98.01%	*Bartonella* sp. in *Meles anakuma* from Japan	CP019788
**58**	*gltA*	99.00%	98.81%	*Bartonella* sp. in *Meles anakuma* from Japan	CP019788
	rpoB	100.00%	98.02%	*Bartonella* sp. in *Meles anakuma* from Japan	CP019788
**59**	*gltA*	100.00%	100.00%	*Bartonella henselae* in cat from Brazil	MN107415
	rpoB	100.00%	99.56%	*Bartonella henselae* in lion (*Panthera leo*) from South Africa	KX499338
**60**	*gltA*	100.00%	100.00%	*Bartonella henselae* in cat from Brazil	MN107415
**61**	*gltA*	100.00%	100.00%	*Bartonella henselae* in fleas removed from cats in Chile	KY913626
	rpoB	100.00%	99.72%	*Bartonella henselae* in cat from Brazil	MN107418
**64**	*gltA*	100.00%	99.82%	*Bartonella henselae* in cat from Brazil	MN107415
**65**	*fstZ*	100.00%	100.00%	*Bartonella henselae* in human from Germany	CP020742
	gltA	100.00%	100.00%	*Bartonella henselae* in fleas removed from cats in Chile	KY913626
	ITS	100.00%	100.00%	*Bartonella henselae* in cheetah (*Acinonyx jubatus)* from Zimbabwe	KX499346
	nuoG	100.00%	99.70%	*Bartonella henselae* in human from Germany	CP020742
	rpoB	100.00%	99.72%	*Bartonella henselae* in lion (*Panthera leo*) from South Africa	KX499338
**67***	*fstZ*	100.00%	100.00%	*Bartonella henselae* in human from Germany	CP020742
	glta	99.00%	98.73%	*Bartonella* sp. in *Meles anakuma* from Japan	CP019788
	ITS	100.00%	100.00%	*Bartonella henselae* in cheetah (*Acinonyx jubatus)* from Zimbabwe	KX499346
	rpoB	100.00%	98.16%	*Bartonella henselae* in cat from Brazil	MN107418
**69**	*gltA*	99.00%	100.00%	*Bartonella henselae* in human from Germany	CP020742
	ITS	100.00%	100.00%	*Bartonella henselae* in flea from Iran	KT314216
	nuoG	100.00%	99.72%	*Bartonella henselae* in human from Germany	CP020742
	rpoB	100.00%	99.73%	*Bartonella henselae* in cat from Brazil	MN107418
**70**	*gltA*	100.00%	100.00%	*Bartonella henselae* in cat from Brazil	MN107415
	rpoB	100.00%	99.72%	*Bartonella henselae* in cat from Brazil	MN107418
**71***	*fstZ*	100.00%	100.00%	*Bartonella henselae* in human from Germany	CP020742
	gltA	100.00%	97.80%	*Bartonella henselae* in fleas removed from cats in Chile	KY913626
	ITS	100.00%	100.00%	*Bartonella henselae* in cheetah (*Acinonyx jubatus)* from Zimbabwe	KX499346
	nuoG	100.00%	99.71%	*Bartonella henselae* in human from Germany	CP020742
	rpoB	100.00%	98.15%	*Bartonella* sp. in *Meles anakuma* from Japan	CP019788

***Co positive sample**

Table S4: Analysis of identity performed in BLAST for flea samples

Sample	Gene	Query Cover	Identity %	Identity/host/country/GenBank accession number	
**7***	*fstZ*	100.00%	99.81%	*Bartonella henselae* in human from Germany	CP020742
	glta	100.00%	98.37%	*Bartonella* sp. in *Meles anakuma* from Japan	CP019788
	ITS	100.00%	100.00%	*Bartonella henselae* in cat from Brazil	MT095053
	rpoB	100.00%	98.12%	*Bartonella* sp. in *Meles anakuma* from Japan	CP019788
**12***	ITS	100.00%	100.00%	*Bartonella henselae* in cat from Brazil	MT095053
	rpoB	100.00%	97.98%	*Bartonella* sp. in *Meles anakuma* from Japan	CP019788
**16***	*fstZ*	100.00%	99.82%	*Bartonella henselae* in human from Germany	CP020742
	rpoB	100.00%	98.01%	*Bartonella* sp. in *Meles anakuma* from Japan	CP019788
**20**	*fstZ*	100.00%	99.91%	*Bartonella henselae* in human from Germany	CP020742

	gltA	100.00%	99.87%	*Bartonella henselae* in fleas removed from cats in Chile	KY913627
	ITS	100.00%	100.00%	*Bartonella henselae* in cat from Brazil	MT095053
	nuoG	100.00%	99.68%	*Bartonella henselae* in human from Germany	CP020742
	rpoB	100.00%	99.72%	*Bartonella henselae* in cat from Brazil	MN107418
**21**	ITS	100.00%	100.00%	*Bartonella henselae* in cat from Brazil	MT095053
**24**	*gltA*	100.00%	100.00%	*Bartonella henselae* in cat from Brazil	MN107415
**35**	*rpoB*	100.00%	100.00%	*Bartonella henselae* in cat from Brazil	MN107418
**37**	*rpoB*	100.00%	98.00%	*Bartonella* sp. in *Meles anakuma* from Japan	CP019788
	fstZ	100.00%	99.81%	*Bartonella henselae* in human from Germany	CP020742
	gltA	100.00%	100.00%	*Bartonella henselae* in cat from Brazil	MN107415
**38**	ITS	100.00%	100.00%	*Bartonella henselae* in cat from Brazil	MT095053
	nuoG	100.00%	99.43%	*Bartonella henselae* in human from Germany	CP020742
	rpoB	100.00%	99.72%	*Bartonella henselae* in cat from Brazil	MN107418
**42**	*rpoB*	100.00%	97.99%	*Bartonella* sp. in *Meles anakuma* from Japan	CP019788
	fstZ	100.00%	100.00%	*Bartonella henselae* in human from Germany	CP020742
**50**	*gltA*	100.00%	99.64%	*Bartonella henselae* in cat from Brazil	MN107415
	ITS	100.00%	100.00%	*Bartonella henselae* in cat from Brazil	MT095053
	gltA	99.00%	98.81%	*Bartonella* sp. in *Meles anakuma* from Japan	CP019788
**51**	ITS	100.00%	100.00%	*Bartonella* sp. in flea from Saint Kitts and Nevis	MT048286
	nuoG	100.00%	96.78%	*Bartonella* sp. in *Meles anakuma* from Japan	CP019788
	rpoB	100.00%	98.01%	*Bartonella* sp. in *Meles anakuma* from Japan	CP019788
	gltA	100.00%	98.77%	*Bartonella* sp. in *Meles anakuma* from Japan	CP019788
**52**	ITS	100.00%	100.00%	*Bartonella* sp. in flea from Saint Kitts and Nevis	MT048286
	nuoG	100.00%	96.76%	*Bartonella* sp. in *Meles anakuma* from Japan	CP019788
	rpoB	100.00%	98.01%	*Bartonella* sp. in *Meles anakuma* from Japan	CP019788
	gltA	100.00%	98.88%	*Bartonella* sp. in *Meles anakuma* from Japan	CP019788
**54***	ITS	100.00%	100.00%	*Bartonella henselae* in cat from Brazil	MT095053
	nuoG	100.00%	96.75%	*Bartonella* sp. in *Meles anakuma* from Japan	CP019788
	rpoB	100.00%	98.01%	*Bartonella* sp. in *Meles anakuma* from Japan	CP019788
**59**	*rpoB*	100.00%	99.72%	*Bartonella henselae* in cat from Brazil	MN107418

**Co positive sample*
